# Tuberculosis diagnosis and management in the public versus private sector: a standardised patients study in Mumbai, India

**DOI:** 10.1136/bmjgh-2022-009657

**Published:** 2022-10-19

**Authors:** Benjamin Daniels, Daksha Shah, Ada T Kwan, Ranendra Das, Veena Das, Varsha Puri, Pranita Tipre, Upalimitra Waghmare, Mangala Gomare, Padmaja Keskar, Jishnu Das, Madhukar Pai

**Affiliations:** 1 McCourt School of Public Policy, Georgetown University, Washington, District of Columbia, USA; 2 Public Health Department, Municipal Corporation of Greater Mumbai, Mumbai, India; 3 School of Medicine, University of California San Francisco, San Francisco, California, USA; 4 Institute for Socio-Economic Research on Development and Democracy, Delhi, India; 5 Department of Anthropology, Johns Hopkins University, Baltimore, Maryland, USA; 6 McGill International TB Centre, McGill University, Montreal, Québec, Canada

**Keywords:** tuberculosis, public health, infections, diseases, disorders, injuries, cross-sectional survey

## Abstract

**Background:**

There are few rigorous studies comparing quality of tuberculosis (TB) care in public versus private sectors.

**Methods:**

We used standardised patients (SPs) to measure technical quality and patient experience in a sample of private and public facilities in Mumbai.

**Results:**

SPs presented a ‘classic, suspected TB’ scenario and a ‘recurrence or drug-resistance’ scenario. In the private sector, SPs completed 643 interactions. In the public sector, 164 interactions. Outcomes included indicators of correct management, medication use and client experience. Public providers used microbiological testing (typically, microscopy) more frequently, in 123 of 164 (75%; 95% CI 68% to 81%) vs 223 of 644 interactions (35%; 95% CI 31% to 38%) in the private sector. Private providers were more likely to order chest X-rays, in 556 of 639 interactions (86%; 95% CI 84% to 89%). According to national TB guidelines, we found higher proportions of correct management in the public sector (75% vs 35%; (adjusted) difference 35 percentage points (pp); 95% CI 25 to 46). If X-rays were considered acceptable for the first case but drug-susceptibility testing was required for the second case, the private sector correctly managed a slightly higher proportion of interactions (67% vs 51%; adjusted difference 16 pp; 95% CI 7 to 25). Broad-spectrum antibiotics were used in 76% (95% CI 66% to 84%) of the interactions in public hospitals, and 61% (95% CI 58% to 65%) in private facilities. Costs in the private clinics averaged rupees INR 512 (95% CI 485 to 539); public facilities charged INR 10. Private providers spent more time with patients (4.4 min vs 2.4 min; adjusted difference 2.0 min; 95% CI 1.2 to 2.9) and asked a greater share of relevant questions (29% vs 43%; adjusted difference 13.7 pp; 95% CI 8.2 to 19.3).

**Conclusions:**

While the public providers did a better job of adhering to national TB guidelines (especially microbiological testing) and offered less expensive care, private sector providers did better on client experience.

**What is already known on this topic**

- There are few studies directly comparing public and private sector quality of medical care using rigorous methods.
- India has the world’s highest burden of tuberculosis (TB), and although free care is available in the public sector, over half of all patients with TB seek care in the private sector.
- This study used simulated standardised patients (SPs) presenting TB scenarios in the public and private sectors of Mumbai, India, to accurately measure and compare practice between sectors across a wide range of outcomes.

**What this study adds**

- The researchers sent SPs presenting two TB case scenarios to 393 private sector facilities and 63 public sector facilities, completing 807 provider-patient interactions.
- Private sector providers were more likely to use chest X-rays for diagnostic purposes, while public sector providers were more likely to use sputum-based testing, in line with governmental guidelines. Both sectors extensively prescribed antibiotics.
- Private sector providers had significantly higher fees than public providers; private providers also offered shorted waiting times, longer consultations and more patient-centric care.

**How this study might affect research, practice or policy**

- In terms of proportions of patients who were correctly managed, the public sector performed more microbiological testing, while the private sector performed more X-ray screening. On metrics of patient-centric and procedural care, such as the time spent with the patient or the completion of checklists, the private sector performed slightly better.
- Since the public sector offers higher use of microbiological tests, is more consistent with national standards, and is free, one potential quality improvement approach is to increase patient demand for public TB services to meet capacity. One way to do this would be to focus more on patient-centred care in terms of time spent with clients, better history-taking and counselling. The other approach is to work with the private sector to increase microbiological testing, and reduce patient costs, via private provider engagement programmes. Both approaches are already being used in Mumbai and other parts of India.

## Introduction

That universal health coverage in low-income and middle-income countries (LMICs) requires not just coverage or utilisation, but also access to high-quality healthcare is increasingly recognised.^1–3^ As multiple studies on quality of care show, those seeking care for common conditions ranging from malaria to asthma to diarrhoea are unlikely to be appropriately diagnosed or managed, with underuse of tests and overuse of medicines, especially antibiotics.^4–8^

Particularly challenging is the intersection between quality of healthcare and the prominent private sector in many resource-poor settings.^9^ Several observers have argued that quality of care is particularly deficient in the private sector, raising challenging questions of why patients continue to pay out-of-pocket for visits to private or informal sector providers when the public sector offers a seemingly viable and lower cost alternative.^10–13^ Indeed, during the COVID-19 crisis, patients have relied heavily on the public healthcare sector, even as the private sector has struggled.^14^

Given the critical policy questions that it raises, it is surprising that the evidence on quality differences between public and private sector care is not particularly robust. Ideally, we would compare ‘like for like’, asking how the *exact same patients* are treated when they visit providers in the public sector compared with the private sector. Another approach would compare the same patient *seeing the same doctor* in their public and private sector locations, allowing researchers to isolate the sectoral component of a given provider’s care decisions (as opposed to potential differences in which providers practise in the public vs private sector).

Of the multiple studies on private and public sector care, only one study from rural India compared quality for the same patient and provider.^15 16^ This study showed large sectoral effects on provider behaviour: the same provider was more likely to appropriately manage a standardised patient (SP) in their private compared with their public sector clinic, with no difference in the use of medicines or antibiotics.

We now provide the first public-private comparison for urban India, using tuberculosis (TB) as a lens through which to examine differences in quality of care across public and private sector providers in the city of Mumbai. The study builds on a previous large study that looked at the diagnosis and management of TB in Mumbai’s private sector,^17^ and we establish a comparison by using the *same* individual SPs and case scenarios in the public sector. Although we do not compare the same doctor in their public and private clinics, we are able to isolate multiple features of TB management from the patient perspective.

Recent research based on large-scale studies of the Indian healthcare system has shown that the country’s private healthcare system is a large, diverse environment.^18^ As much as 70% of healthcare is sought from private primary care providers nationwide. In Mumbai, street-by-street mappings of healthcare providers have uncovered a large and varied private sector, ranging from highly trained and specialised chest physicians to providers who are trained in alternative systems of medicine, but who also diagnose and treat allopathically (the main recognised systems are Ayurveda, Yoga, Unani, Siddha and Homeopathy, known collectively as AYUSH providers).^19^ Not surprisingly, the predominance of the private sector extends to the diagnosis and management of TB: A recent study estimated that private provision of TB medication accounts for two-thirds of the national supply, dispensing around 18 million patient-month supplies annually compared with the 9 million or so delivered by the public sector each year.^20^

Our decision to use TB as an index case was based on two related factors. First, the previous study in rural India studied public-private differences for quality of care used angina, asthma and childhood diarrhoea.^16^ While there are important public health considerations here such as the overuse of antibiotics, the first-order costs of poor diagnosis and management for these common conditions are primarily borne by the patient and their family. In contrast, TB is airborne and contagious, and therefore poor diagnoses pose additional risks to the broader population in addition to potentially devastating consequences for the afflicted. This contagion externality implies that there is a potential gap between the individually optimal care for a given patient and the public health rationale for specific types of management, and this gap may become apparent in the comparison of public versus private sector care.

Second, until the COVID-19 pandemic, TB was the largest single infectious disease cause of mortality worldwide. Mumbai has a high burden of TB, including drug-resistant TB.^21 22^ The continuing high prevalence of TB in the population and the emergence of increasingly drug-resistant strains has led the Government of India to take a more aggressive stance towards the disease with the Ministry of Health committing to elimination of the disease by 2025.^23^ The Mumbai Mission for TB Control was launched in 2013, and is led by the Municipal Corporation of Greater Mumbai (MCGM), the city government. The greater focus on TB within the public sector implies that we are able to provide estimates of quality of care for a disease that a disease that is critical both from epidemiological and political perspectives: TB had considerable political will and resources dedicated to its control, including the engagement of private providers in the city.^24^

To measure the quality of care in the public and private sector, we used SPs—trained members of the community who present the same case to multiple providers posing as patients. This method has gained rapid acceptance in quality measurement in countries around the world.^13 25 26^ The use of SPs is informed by the fact that the most easily observable characteristics of facilities—including facility equipment, provider education levels, medication supplies and patient access—are poor predictors of key quality metrics.^27^ The research community has developed and validated SP methods for directly observing quality of care in multiple primary care settings.^2 28^ The SP method used here allows researchers to compare the care that was provided against predetermined national and international standards of care, avoids Hawthorne effects whereby providers change their behaviour because they know they are being observed, and allows researchers to control for potential confounding arising from differences in case-mix and patient-mix across healthcare providers. Using this method in a representative sample of public facilities in Mumbai, we compare the management of SPs in the public sector with the management of SPs presenting identical cases in private sector facilities.

## Methods

### Public and private health systems in Mumbai

Primary care in Mumbai is available through both public and private sector clinics. Patients choosing to visit publicly funded facilities have a range of options from tertiary hospitals (usually attached to medical colleges), smaller, peripheral hospitals, to primary health centres and dispensaries staffed with qualified doctors. Doctors in the public sector receive a fixed salary, and all consultations, lab tests and medicines should be free. Costs are standardised across the city in the public sector. While tertiary, public hospitals often have specialists (eg, chest physicians), primary care centres are typically staffed by MBBS trained doctors, pharmacists, nurses and other staff.

Patients choosing the private sector have a range of options, from informal providers, to AYUSH practitioners, to qualified MBBS general practitioners, as well as specialists in secondary and tertiary care centres and hospitals. Cost of consultation varies widely across this spectrum, with informal and AYUSH providers being the most affordable, and specialists in private and corporate hospitals being the most expensive. Costs in the private sector are not standardised or regulated for most part.

### The SP methodology

SPs are trained community members who pose as actual patients seeking care in a healthcare provider’s place of practice. They are recruited from local communities, speak the local language and are extensively trained to portray a predetermined and scripted medical condition to those healthcare providers, as well as to recall accurately and in great detail the actions of the provider.^13^ Previous publications provide more details on the SP methodology,^12 25 29–31^ and prior studies using the method for TB in India, China, Kenya and South Africa have demonstrated the validity of the method for TB presentation with no significant risks to providers, SPs or other patients.^12 17 26 28 30 32–34^

Informed by clinical observation,^35^ all SP interactions start with an opening statement, which is the primary presenting complaint from the patient. Table 1 lists the initial symptoms that our two SP scenarios reveal in their complaint to the provider. Doctors then proceed exactly as they do with any other patients—after all, they have no reason to doubt the veracity of the case presentation.

**Table 1:** Standardised patient scenarios and expected correct management

	Case 1	Case 2
Scenario description	Classic, suspected TB	Recurrence or drug resistance
Case description	Classic case of presumed TB with 2–3 weeks of cough and fever	Chronic cough, and, if asked, elaborates a history of previous incomplete treatment for TB, which would raise the suspicion of drug resistance.
Patient presentation	Presents with presumptive TB, for the first time, to the provider, saying: “Doctor, I have cough that is not getting better and some fever too.”	Presents as a previously treated for TB with recurrence of the disease, saying, “Doctor, I am suffering from a bad cough. One year ago, I had got treatment in (the local public hospital), and it had got better. But now I am having cough again.”
STCI management	Recommendation for sputum testing, chest radiograph or referral to a public DOTS centre or provider	Recommendation for any drug-susceptibility test (culture, line probe assay, or Xpert MTB/RIF), or referral to a public DOTS centre or provider
RNTCP guidelines	Sputum smear examination to be used as the frontline test. Drug-susceptibility test (culture, line probe assay, or Xpert MTB/RIF) acceptable but not required except for confirmed TB or special populations.	Sputum smear examination to be used as the frontline test. Drug-susceptibility test (culture, line probe assay, or Xpert MTB/RIF) acceptable but not required except for confirmed TB or special populations.
Sampling: private	1x at 393/393 purposively sampled facilities	1x at 250/393 purposively sampled facilities
Sampling: public dispensaries	1x at 44 randomly sampled of 175 listed facilities	1x at 44 randomly sampled of 175 listed facilities
Sampling: public hospitals	2x at each of the available 19 facilities	2x at each of the available 19 facilities

RNTCP, Revised National TB Control Programme; STCI, Standards for TB Care in India; TB, tuberculosis.

Extensive work on specific case histories ensure that the answers that SPs give help drive the provider towards the TB diagnosis. Therefore, there is an extensive list of history questions that the SP will answer in the affirmative, as well as other questions that the SP will say ‘no’ to. For instance, if the doctor asks whether the SP has experienced a productive cough (not dry), weight loss or night sweats, the SP will say ‘yes’. SPs are also trained to reply in the negative to questions that serve to rule out symptoms of other potential causes, such as asthma and allergies.

### Data

For this study, we used SP data collected from a project designed to assess the quality of TB care among public and private healthcare providers in 2019 in Mumbai. The study was conducted in 2018 and 2019 at city-wide scale in both the public and private sectors in Mumbai, allowing us to estimate accurate comparative measures of the way in which patients presenting in the private and public sectors are initially managed. A full description of the study design and results from an earlier study round (conducted in 2014–2015) in the private sector has previously been published.^31^ This study reports results using new data collected several years later among the same sample, combined with an additional sample of public sector facilities in Mumbai. Our detailed descriptions and comparisons of the behaviour of facilities across sectors is further enabled by the fact that the same individual SPs were used in each case to present identical TB case scenarios in both sectors, and the SPs had been specifically trained to report a wide range of outcomes through a structured questionnaire.

In that project, 11 SPs visited 393 different randomly sampled private healthcare facilities between 28 September 2018 and 16 January 2019 (completing 644 interactions). Private sector providers in this study were providers with a Bachelor of Medicine, Bachelor of Surgery (MBBS) degree, or specialists with an additional MD (typically in chest medicine). Stratified sampling was used to randomly oversample providers enrolled in private provider engagement TB programmes in the city; overall, the sample was broadly representative of a wide geography in the city’s private sector. A complete description of these samples is available in prior publication.^17^

New data collection on the public sector was also conducted in Mumbai.^36^ In the public sector, SPs visited a random sample of 25% of the city’s 175 public dispensaries (N=44) and all of the 15 peripheral hospitals and four medical colleges (reported together as ‘public hospitals’) operated by the Municipal Corporation of Greater Mumbai (MCGM). SPs successfully completed interactions at all 44 randomly sampled public sector dispensaries and the 19 public hospitals, for a total of 164 interactions at 63 different public healthcare facilities between 25 January 2019 and 14 March 2019. Online supplemental appendix figure A5 verifies the validity of the random sample of dispensaries by reporting the results of a comparison of TB-related administrative statistics in sampled dispensaries relative to the complete listing of dispensaries using MCGM administrative data. We found no detectable statistical differences along any margin, which confirmed the representativeness of the public sector facility sample.

10.1136/bmjgh-2022-009657.supp1 Supplementary data

Across these facilities, we deployed SPs presenting with two predetermined TB case scenarios. The two SP cases developed with the support of a technical advisory group comprised of clinicians, public health experts, economists, anthropologists, and relying on both the Standards for TB Care in India (STCI) and the International Standards of TB Care.^37 38^ In case 1, a ‘classic, suspected TB’ profile of a person who should be evaluated for TB, the SP presented with 2 weeks of cough and fever and revealed additional characteristic symptoms suggestive of TB disease if questioned. In case 2, a ‘recurrent/drug-resistant’ profile, the SP also presented with 2 weeks of cough, and further explained that these symptoms were recurrent from a previous episode in which TB was diagnosed and treated, but that the treatment regimen had been discontinued when their symptoms had improved. The presentation was intended to be highly suggestive of recurrent and potentially drug-resistant TB. The details of the two SP cases and their assignment are presented in table 1; the full case scenarios, demographic backgrounds, and the pre-scripted answers to a range of anticipated history questions are included in the online supplemental material.

In the private sector, 394 case 1 interactions and 250 case 2 interactions were completed. In the public sector, 82 case 1 interactions and 82 case 2 interactions were completed. In total, 11 SPs (4 women and 7 men) portrayed those cases. Each SP portrayed only one of the two cases at a time, and only one SP presented a different case in the public sector than in the private sector. Three SPs did not participate in the public sector portion and one did not participate in the private sector portion. All medicines prescribed or offered to the SPs were independently coded and classified after being brought to the study office. No subgroup analyses were performed as part of this study. Prior publications examined variation in outcomes across individual standardised patients and by SP gender specifically; both found small or zero effects of SP identity on care outcomes within this sample.^33 34^

### Outcome measures

We divided outcome measures into four ‘families’, which we used to adjust standard errors (SEs) and confidence intervals (CIs) for multiple hypothesis testing. Families 1 and 2 captured the case-specific appropriateness of clinical management (or adherence to technical guidelines). Family 1 included two binary summary indicators of correct management and the share of checklist questions asked, both of which were predetermined, case-specific outcomes.^37^ First, we used the Standards for TB Care in India (STCI) guidelines, consistent with past evaluations of the private sector, which are best practices promulgated but not necessarily enforced by the Ministry of Health & Family Welfare. Second, we also used as a benchmark the Revised National TB Control Programme (RNTCP) guidelines, by which public sector providers are trained and evaluated. RNTCP was recently renamed as National Tuberculosis Elimination Programme (NTEP). We assess both sectors against both measures for comparability. Our rationale for using two standards was that the STCI yardstick allowed us to compare the results with our previous SP studies in India, while the RNTCP standard allowed us to judge public providers on the basis of what they are expected to follow.

Benchmarks for the STCI-based and the RNTCP-based summary indicators of correct management differed due to the fact that prior SP work in India had focused on the private health sector, while the current work also included the public sector. Past studies used an aggregate definition of correct management for each case based on international and local standards.^17 30^ Since this study was also conducted in the public sector, we included an additional benchmark for quality of care for both sectors defined by the then-current public sector diagnostic protocol. That definition followed the 2016 Technical and Operational Guidelines for TB Control in India and the 2017 Guidelines on Programmatic Management of Drug-resistant TB by the RNTCP or NTEP.^39 40^ We report top-line results using both definitions for both sectors, such that all reported direct comparisons compare identically defined indicators across the two sectors.

Previous work defined correct management for case 1 as a recommendation for any sputum testing (sputum smears, Xpert MTB/RIF or culture), or chest radiograph, or referral to a public DOTS centre or a private provider or specialist; for case 2, correct management was defined as recommendation for any drug-susceptibility test (culture, line probe assay or Xpert MTB/RIF) or referral to a public DOTS centre or to a private provider or a specialist, specialist since the patient has recurrent TB symptoms, suggestive of drug-resistance TB.^31^ These definitions reflected the Standards for TB Care in India, and was also used in the previous SP surveys in India, all of which were conducted among private health sector providers.^37^ For the private sector, we used a lenient definition of correct management, and accepted a wide range of TB tests, including chest X-rays (CXRs), which are not confirmatory but acceptable as triage tests.

For case 1, RNTCP or NTEP guidelines recommended that all presumptive TB cases would receive sputum smear examinations as the frontline test. CXRs were not the frontline TB test, since the emphasis in the public sector was sputum-based, microbiological testing. For the following key and vulnerable populations, RNTCP allowed the use of Xpert MTB/RIF (CBNAAT) as the frontline molecular test: paediatric age groups, people living with HIV, extrapulmonary TB sites and smear-negative individuals with X-rays suggestive of TB. Case 1 did not fit any of these special, vulnerable populations. The RNTCP approach for Universal Drug Susceptibility Testing (UDST) allowed for all diagnosed and notified TB patient to later receive Xpert MTB/RIF.^41^ In other words, Xpert testing was allowed after TB was microbiologically diagnosed. So, for case 2, an initial approach of TB diagnosis using sputum testing was acceptable, under these guidelines, since direct use of Xpert MTB/RIF as a frontline test did not cover patients with prior history of TB therapy.

Family 2 outcomes focused on medications and included: starting TB treatment without test results or giving quinolone antibiotics, broad-spectrum antibiotics or steroids.^42 43^ Medications from each interaction were ex-post coded by name to correspond to ATC code classifications. Fluoroquinolone antibiotics were defined as ATC codes beginning with J01M; broad-spectrum antibiotics were all other ATC codes beginning with J01 except anti-TB medications; and steroids were defined as ATC codes beginning with H02, R01 or R03. The previous literature has identified both the use of quinolones and steroids as potentially contributing to drug resistance and diagnostic delays due to symptom and immune response suppression,^44–47^ and there is a persistent concern that the overuse of antibiotics is leading to the proliferation of antibiotic-resistant TB variants in the general population. It is widely believed that the dominance of a poorly regulated fee-for-service, private sector is one of the main reasons for high antibiotic usage in India.^48 49^

Outcomes in family 3 addressed a persistent concern that user experience and patient satisfaction might be suboptimal in the public sector.^50^ We included a large set of quality indicators that captured the experience of the interaction in family 3, ranging from the subjective rating given by the SP on a 1-to-10 scale and whether the SP would visit the provider again to specific questions like whether the provider was distracted (eg, whether they used a cell phone or whether there was a television on at the time of the interaction). While we recognised that SPs are not truly ill and the subjective indicators here could not be translated directly to patient experiences, we included them to provide an assessment of *differences* between the two sectors. SPs, unlike other patients, have the unique ability to visit multiple facilities presenting as the same person with the same complaint at the same stage of treatment—therefore, their perception of their interpersonal treatment in various circumstances is a comparator that is difficult to obtain from other patients (whose experience will necessarily evolve over multiple presentations).

Finally, family 4 included three other cost and convenience measures that are important to understand differences between the two sectors. These were the time the SP spent waiting to see the provider, the amount paid they for the interaction, and the time the provider spent with the SP. Previous studies had identified long wait times and short consultation length as two potential deficits in the public sector.^16 35 50^

### Statistical analysis

To estimate quality levels separately by health sector, we took advantage of the fact that all providers were visited by the same SPs and presented with identically scripted case presentations. Therefore, we treated the mean outcomes for each category of provider as unbiased measures of the average behaviour of the providers visited in that sector for that case presentation. Fieldwork was organised such that the same SPs were used first for the private sector data collection and then for the public sector, meaning that the personal characteristics of SPs were the same across the two sectors. As described in prior work, multilevel outcome structures were taken into account by modelling individual effects for the standardised patients, as well as adjusting CIs for hierarchical clustering within facilities (since multiple visits were conducted at each).^33^

Power calculations were based off completed private sector data collection using two-proportions comparisons. These calculations indicated that, for the observed levels of TB testing and the sample size in the private sector data collection, 80% power would be achieved for a 10-percentage-point difference at maximum 145 observations (considering base rates of 85% or 15%) and minimum 105 observations (considering base rates of 90% or 10%) in the public sector. Therefore, our pooled (unweighted) sample of 164 public sector interactions was sufficiently powered for all two-way comparisons included in the study, and we adjusted CIs for multiple comparisons appropriately as described below.

We used ordinary least squares regression to assess differences in clinical care processes and case management across facilities by sector. In pooled specifications, we controlled for differences across case scenario as well as the individual SP identity. These attributes may affect quality of care and we controlled for them to compare across sectors only within identically scripted patient presentations.^31^ We clustered SEs at the facility level when overall regression differences to avoid overstating the precision of our estimates due to repeated visits with the same providers. Therefore, estimates corresponded to the expected average quality of care outcomes and sectoral differences if facilities were chosen at random from the sampling list for each sector.^31 51^ When multiple related outcomes were regressed simultaneously, statistical significance was determined using the Benjamini-Hochberg procedure to control the false discovery rate at α=0.050.^52^ All data analysis was performed with Stata 17 (StataCorp, Texas, USA).^53^

### Patient and public involvement

No actual patients were involved in this research study, since we used simulated SPs only to collect data on provider behaviour. However, simulated SPs and the research questions they engage are by their nature based on the experience of real patients and their interests. In developing the case scenarios, presentations, medical and social histories, and in developing tools to measure both technical care quality as well as client experience, the team relied heavily on their observations from and work with actual patients throughout their prior work. The involvement of local researchers is described in the author reflexivity statement (see online supplemental appendix).

## Results

### Case management

**Figure 1:**
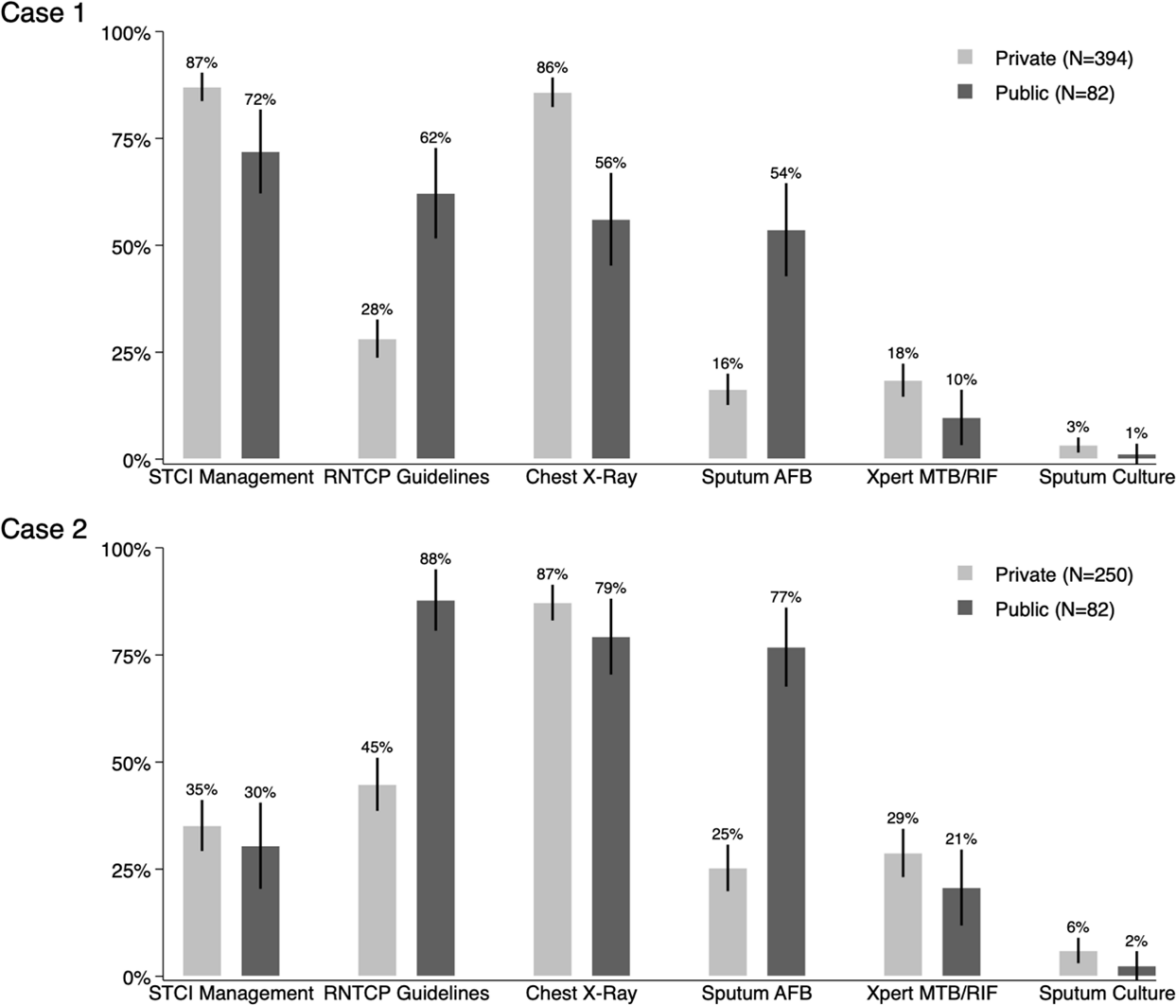
TB testing outcomes by sector. This figure reports a variety of measures for all SP interactions. The STCI management measure is defined as each case 1 interaction that received a referral, a chest X-ray, a sputum AFB test, or an Xpert CBNAAT test or other drug-susceptibility test; and for each case 2 interaction that received a referral or an Xpert CBNAAT test or other drug-susceptibility test. All other measures are as reported by the SP after the interaction was completed. AFB, acid-fast bacillus; TB, tuberculosis; Xpert MTB/RIF, CBNAAT *Mycobacterium tuberculosis*/rifampicin sensitivity testing.

Table 2 reports the outcome measures we used along with summary means for each measure. Figure 1 provides an overview of the use of diagnostic testing by sector and case scenario. In this figure, we pooled all public sector facilities into a single group, and present them alongside the private sector testing outcomes. Most notably, sputum acid-fast bacillus (AFB) smears were the most common microbiological test used in the public sector private sector, while initial management was dominated by use of CXRs in the private health sector. The public sector also offered CXRs for a substantial number of SPs, but did so in fewer interactions than the private sector and in fewer interactions than microbiological tests were used.

**Table 2:** Outcome measures

	Case 1	Private	Public hospital	Public dispensary	Case 2	Private	Public hospital	Public dispensary
Family 1: appropriate clinical management	Mean	N	N	N	Mean	N	N	N
STCI management	0.84	394	38	44	0.34	250	38	44
RNTCP guidelines	0.42	394	38	44	0.36	250	38	44
Share of questions	0.34	394	38	44	0.55	250	38	44
Chest X-ray	0.81	394	38	44	0.85	250	38	44
Sputum AFB	0.23	392	38	44	0.38	249	38	44
Xpert MTB/RIF	0.17	390	38	44	0.27	250	38	44
TB suspicion	0.51	393	38	44	0.55	250	38	44
Referred away	0.02	391	38	44	0.06	250	38	44

Family 2: medication use	Mean	N	N	N	Mean	N	N	N
Started TB treatment	0.00	394	38	44	0.04	250	38	44
Fluoroquinolones	0.05	394	38	44	0.05	250	38	44
Other antibiotic	0.71	394	38	44	0.53	250	38	44
Steroids	0.02	394	38	44	0.03	250	38	44

Family 3: client experience	Mean	N	N	N	Mean	N	N	N
SP Subjective Rating (1–10)	7.44	393	38	44	7.52	249	38	44
Provider used cell phone	0.09	393	38	44	0.11	249	38	44
Other people were in room	0.11	393	38	44	0.08	249	38	44
Provider had a TV on	0.00	393	38	44	0.00	249	38	44
SP liked the provider	0.95	393	37	44	0.95	249	38	44
SP would go to this provider	0.94	393	38	44	0.95	249	38	44
Provider created a private environment	0.84	393	38	44	0.80	249	38	44
Provider seemed knowledgeable about illness	0.60	393	38	44	0.83	249	38	44
Provider addressed worries seriously	0.56	393	38	44	0.60	249	38	44
Provider explained SP condition	0.07	392	38	44	0.09	249	38	44
Provider explained SP treatment plan	0.36	391	38	44	0.36	249	38	44

Family 4: cost and convenience	Mean	N	N	N	Mean	N	N	N
Time waiting (min)	58.36	386	38	44	66.64	249	38	44
Amount paid (INR)	425.25	394	38	44	388.45	250	38	44
Time with provider (min)	4.10	391	38	44	3.88	249	38	44

RNTCP, Revised National TB Control Programme; SP, standardised patient; STCI, Standards for TB Care in India; TB, tuberculosis.

**Figure 2:**
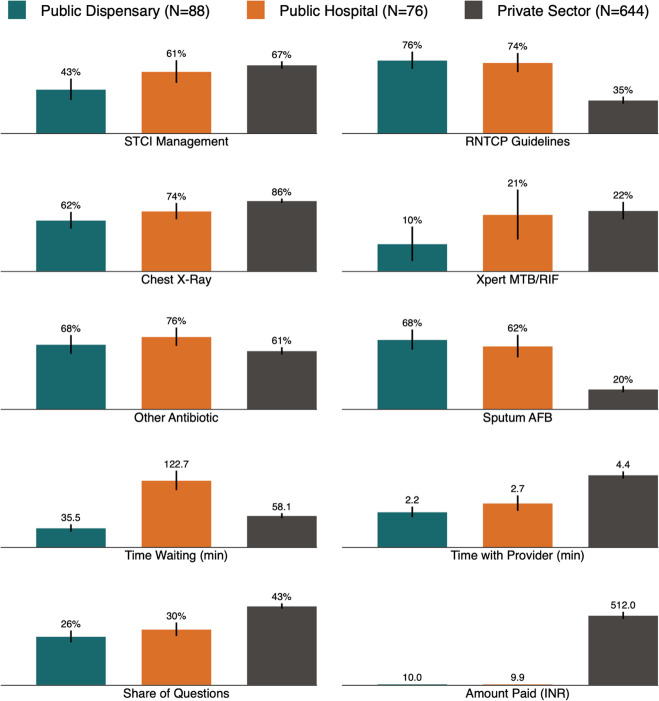
Primary management outcomes by study strata. This figure reports a variety of measures for all SP interactions. The STCI management measure is defined as each case 1 interaction that received a referral, a chest X-ray, a sputum AFB test, or an Xpert CBNAAT test or other drug-susceptibility test; and for each case 2 interaction that received a referral or an Xpert CBNAAT test or other drug-susceptibility test. All other measures are as reported by the SP after the interaction was completed. AFB, acid-fast bacillus;TB, tuberculosis, Xpert MTB/RIF, CBNAAT *Mycobacterium tuberculosis*/rifampicin sensitivity testing.

**Figure 3:**
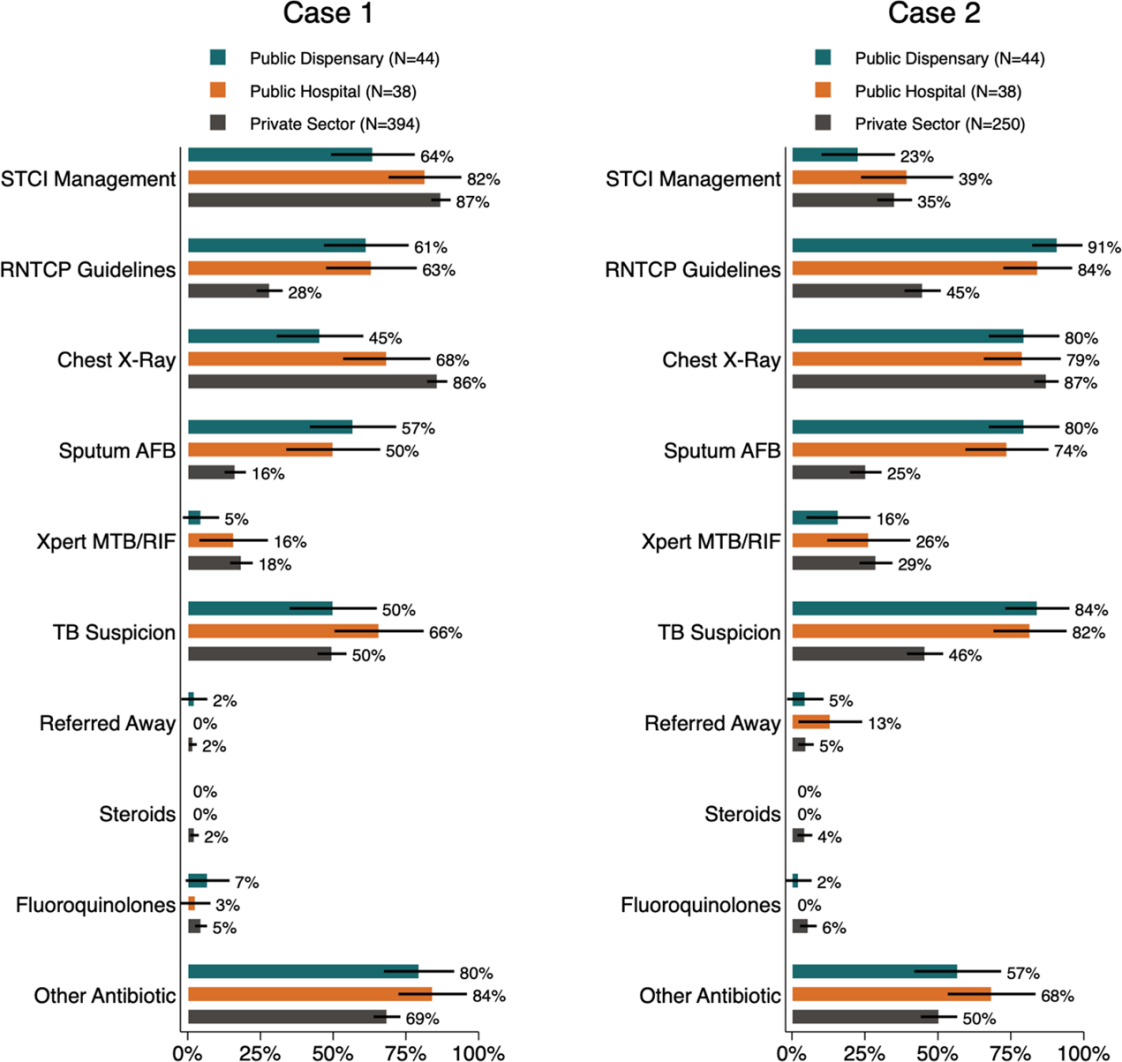
Clinical management by study strata and case. This figure reports the proportion of interactions in each study strata that resulted in the indicated outcome for an SP presenting case 1 or case 2 as indicated. The STCI management measure is defined as each case 1 interaction that received a referral, a chest X-ray, a sputum AFB test, or an Xpert CBNAAT test or other drug-susceptibility test; and for each case 2 interaction that received a referral or an Xpert CBNAAT test or other drug-susceptibility test. AFB, acid-fast bacillus; TB, tuberculosis; Xpert MTB/RIF, CBNAAT *Mycobacterium tuberculosis*/rifampicin sensitivity testing.

We present more detailed breakdowns of non-diagnostic elements and our STCI-based measure of care alongside additional quality characteristics in the next two figures. Figure 2 reports pooled estimates of the quality of care for the classic suspected TB scenario (case 1) and the recurrent TB/drug-resistant scenario (case 2) together, and figure 3 presents a detailed breakdown of management behaviours for each case.

As figure 3 shows, in both cases, public sector providers made more frequent use of microbiological testing, in line with RNTCP protocol (sputum AFB, culture DST or Xpert MTB/RIF). In contrast, private providers were more likely to order a CXR and Xpert MTB/RIF in both cases, doing so in 144 of 639 interactions (23%; 95% CI 19% to 26%). In both cases, public providers were more likely to mention a suspicion of TB to the patient (67% vs 49%; adjusted difference 18%; 95% CI 9% to 27%). Thus, when we defined correct management to be in line with RNTCP guidelines, where the sputum tests were required in both cases first, we found substantially higher proportions of correct management in the public compared with the private sector (75% vs 35%; adjusted difference 35 pp; 95% CI 25 to 46).

When we classified correct management according to previous studies of the private sector (which accepted use of CXRs as an initial test), the results showed that a higher proportion of interactions were correctly managed in the private sector (67%; 95% CI 63% to 70%). This was largely due to the higher rate of CXR use in the private sector.

The private sector itself included doctors with differing qualifications as our private sector sample included a number of specialists with advanced degrees (which we define as holding an MD in addition to the standard MBBS qualification). Online supplemental appendix figure A1 shows that private sector providers with advanced degrees were indeed more likely than other private sector providers to order appropriate microbiological tests, but did not differ substantially on other dimensions of care. (Online supplemental appendix figure A2 and A3 provide detailed comparisons.)

### Cost and convenience

Like the difference in the use of microbiological testing, other measures also suggested stark differences between the types of patient-centric care delivered across the two sectors. Patients spent significantly longer waiting in the public hospitals—about 2 hours—compared with private facilities, where they waited about an hour (57 min vs 126 min; adjusted difference 70 min; 95% CI 34 to 106). Public dispensaries typically had patients wait only 36 min (95% CI 29 to 42). Costs to patients in the public sector were INR 10 with no variation (one visit recorded zero cost paid), while costs to patients in the private clinics averaged INR 512 (95% CI 485 to 539). In further measures of patient experience, we observed two substantial differences between public hospitals and private facilities. First, private providers spent more time with patients than public hospitals (4.4 min vs 2.4 min; adjusted difference 2.0 min; 95% CI 1.2 to 2.9), and, second, private providers asked a correspondingly higher share of questions about the patient’s condition (29% vs 43%; adjusted difference 13.7 pp; 95% CI 8.2 to 19.3).

### Use of medications

The use of medications was broadly similar across all groups of facilities, with very low recorded usage of TB treatment, quinolone antibiotics or steroids. However, the use of other broad-spectrum antibiotics remained high in all settings, with public hospitals offering such medications at 58 of 76 interactions (76%; 95% CI 66% to 84%), and private facilities less likely at 395 of 643 interactions (61%; 95% CI 58% to 65%). We further observed very few providers in any group who chose to refer the patient for outside care. Just 8 of 473 case 1 interactions resulted in referral (2%; 95% CI 1% to 3%), and 19 of 332 case 2 interactions did (6%; 95% CI 4% to 9%); public hospitals were the most likely to do so (13%; 95% CI 6% to 27%). All public sector referrals were within the public sector (primarily to DOTS centres), while two-thirds of private sector referrals were to private specialists and one-third were to the public sector. Since referrals are nearly non-existent in our data, the remaining estimates of clinical management—the diagnostic decisions observed at the visited clinics themselves—are estimates of the complete services that any patient would have received unless they chose to seek further care themselves or return for follow-up care. For both cases, we accept any referral as correct management, and this decision does not affect any main results.

### User experience and patient satisfaction

**Figure 4:**
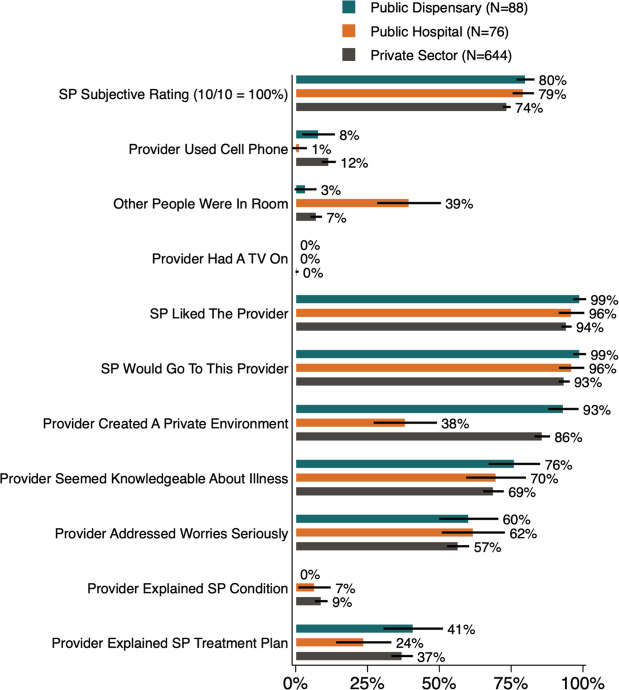
Client experience by study strata. This figure reports a variety of measures that capture the subjective satisfaction of the standardised patient (SP) with the clinical interaction. The first three (cell phone use, other people present and TV on) are factual reports of the provider’s conduct. The measures of whether the SP liked the provider and would see that provider again (personally) are yes/no questions posed to the SP. The remaining questions regarding the SP’s perception of the provider’s conduct require the SP to choose between ‘Not at All’, ‘Somewhat’ and ‘Definitely’; reported here is the share of SPs who responded the provider ‘Definitely’ met the description.

Figure 4 presents differences in the satisfaction and experience reported by the SPs themselves. Prior evidence has suggested cases of suboptimal patient experience, with reports of poor interpersonal treatment of patients in some cases.^50^ We found little evidence of systematic problems with the quality of personal treatment. We also did not observe any substantial gap in SPs’ personal assessments of the experience between the public and private sector. For instance, between 94% and 99% of SPs reported they liked the doctor and would go there again, in both sectors. The only large differences observed were that public hospitals were substantially more likely to have other people in the room with the patient (39% vs 7%; adjusted difference 33 pp; 95% CI 26 to 40). SPs were correspondingly less likely to report that public providers had created a private environment for them (38% vs 86%; adjusted difference 47 pp; 95% CI 36 to 59).

### Regression comparisons by public facility type

**Figure 5:**
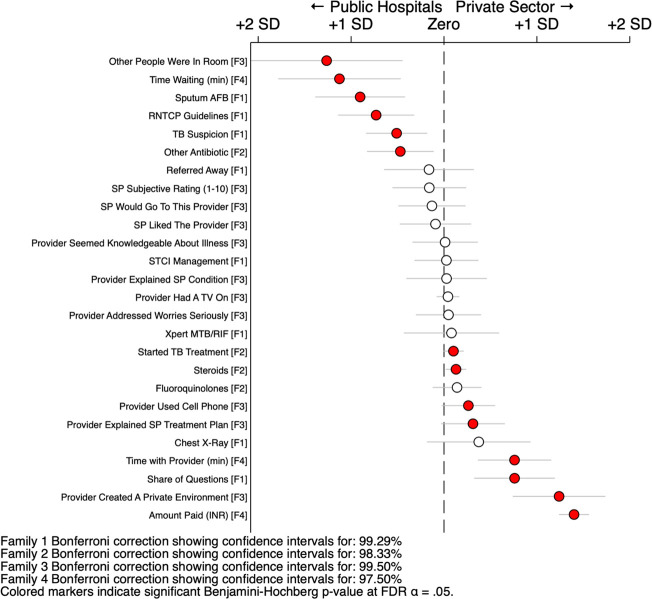
Standardised regression comparison of public hospitals and private sector. This figure reports the results of a series of regressions using both the public hospital interactions (N=76) and private hospital interactions (N=633). Each point reports the coefficient for the indicator variable of the private sector and the corresponding CI. All measures are standardised to mean 0 and SD 1 for illustration purposes. Estimates are controlled for individual standardised patient (SP) ID and the case scenario; SEs are clustered by facility. The measures are grouped into four families for further error correction: family 1 are the appropriate clinical management outcomes; family 2 are the medication use outcomes; family 3 are the subjective experience measures; and family 4 are the unassociated characteristics. Each family’s CIs are extended to the appropriate Bonferroni interval for the number of simultaneous hypothesis; and the p values are considered significant if they satisfy the Benjamini-Hochberg step-up procedure (including values that may not be considered significant under their own Bonferroni interval). Measures are sorted by the magnitude and direction of the estimated coefficient. The STCI management measure is constructed as 1 for each case 1 interaction that received a referral, a chest X-ray, a sputum AFB test, or an Xpert CBNAAT test or other drug-susceptibility test; as 1 for each case 2 interaction that received a referral or an Xpert CBNAAT test or other drug-susceptibility test; and 0 otherwise.

**Figure 6:**
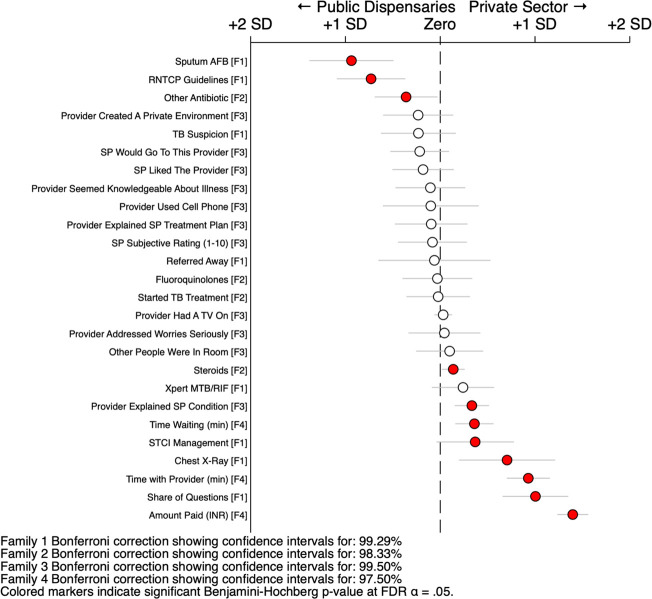
Standardised regression comparison of public dispensaries and private sector. This figure reports the results of a series of regressions using both the public dispensary interactions (N=88) and private hospital interactions (N=633). Each point reports the coefficient for the indicator variable of the private sector and the corresponding CI. All measures are standardised to mean 0 and SD 1 for illustration purposes. Estimates are controlled for individual standardised patient (SP) ID and the case scenario; SEs are clustered by facility. The measures are grouped into four families for further error correction: family 1 are the appropriate clinical management outcomes; family 2 are the medication use outcomes; family 3 are the subjective experience measures; and family 4 are the unassociated characteristics. Each family’s CIs are extended to the appropriate Bonferroni interval for the number of simultaneous hypothesis; and the p values are considered significant if they satisfy the Benjamini-Hochberg step-up procedure (including values that may not be considered significant under their own Bonferroni interval). Measures are sorted by the magnitude and direction of the estimated coefficient. The STCI management measure is constructed as 1 for each case 1 interaction that received a referral, a chest X-ray, a sputum AFB test, or an Xpert CBNAAT test or other drug-susceptibility test; as 1 for each case 2 interaction that received a referral or an Xpert CBNAAT test or other drug-susceptibility test; and 0 otherwise. AFB, acid-fast bacillus; STCI, Standards for TB Care in India.

To summarise these differences, we report standardised regression estimates of differences between private facilities and public hospitals in figure 5, and between private facilities and public dispensaries in figure 6, including all the measures reported previously. All measures were standardised to the same scale by transforming the dependent variable to mean zero and SD 1; they were grouped into four families for purposes of multiple hypothesis correction using Bonferroni CIs. Regressions were then modelled for multilevel data with individual effects for SP and case scenario, with clustering adjustments by facility; additional details are presented in the online supplemental appendix. The figure highlights all statistically significant differences after applying the family-wise Benjamini-Hochberg false discovery rate control procedure.^52^

Compared with public hospitals, private providers charged more money, but spent more time with the SPs; asked more history questions; were more likely to create a private environment; and were more likely to complete more checklist examination and history-taking items. The public providers charged little or nothing; were more likely to have other people in the room; had the SPs wait longer; were more likely to order a sputum TB tests; and were more likely to inform the SP of a suspicion of TB. When correct management was judged by use of sputum-based microbiological testing, then the public sector much fared better. In the equivalent comparison between private facilities and public dispensaries, the public sector dispensaries were also more likely to order sputum-based microbiological tests. The private sector was again more likely to explain the condition, order an X-ray and spend slightly more time and ask more questions. Online supplemental appendix table A2 reports the complete statistics for the estimated differences of all comparisons in linear levels.

## Discussion

Many comparisons between public and private healthcare rely on administrative data from both sectors to draw conclusions. Because these data are scarce and because different sectors even in the same location have different client mixes and different data collection protocols, there are nearly no ‘apples to apples’ comparisons of the quality of care between sectors that use valid counterfactuals.^15^ These statistical issues are unavoidable in administrative data,^25^ leading to literatures that produce extremely heterogeneous results in meta-analysis^54^ or that rely on intermediate indicators rather than quality outcomes.^55^

Only very recent studies—such as one conducted with standardised patients in Xi’an, China, among 212 public providers and 27 private providers—have begun to directly compare equivalent quality outcomes across sectors with gold standard measurement methods.^56^ This study collected data on quality using the method of SPs, similar to a prior study that compared the performance of individual providers in the public and private sectors (when they worked in both).^16^ In that study, a selected subsample of providers and the same (standardised) patients were observed in different contexts, allowing the authors to estimate the effect of the public sector setting on provider performance.

This study observed a fixed sample of the same (standardised) patients in interactions with the true provider mix in each sector, allowing us to estimate differences in the expectations a fully informed individual would have before choosing where to seek care. We do not need to interpret these regressions as estimating a comparison of the care received by the patients who chose each sector; care-seekers would be able to choose between sectors based on their needs and the costs associated with each. In this context, instead, such regression comparisons provide insights into which preferences or needs would lead people to seek care in each sector. This perspective leads to conclusions that cannot be drawn from patient data, because it is designed to analyse outcomes from the options that patients do not actually take up.

There is a widespread view that the public sector in India provides poor quality care, which in turn has led patients towards the private sector.^16 57^ Our study shows that such generalities hide substantial variation *within* the public sector and across outcome measures. In terms of proportions of patients who were correctly managed, the public sector in Mumbai performed much better with microbiological testing, and adherence to national standards. For case 1, the public sector relied on microbiological tests while the private sector preferred CXR; for case 2, public sector providers, consistent with RNTCP guidelines, asked for a sputum test first. We find that on metrics of patient-centric and procedural care, such as the time spent with the patient of the completion of checklists, the private sector seems to perform slightly better.

Furthermore, we did not find substantial differences between the public and private sector in reported patient experience—overall SP ratings were identical across all sectors as were behaviours on specific index outcomes, such as cellphone use during the patient interaction. Both public and private sectors had low use of fluoroquinolones and steroids but similarly high use of broad-spectrum antibiotics. This is consistent with the previous study using SPs in the public and private sector in India.^16^

One interpretation is that care in these two sectors is differentiated by the extent to which doctors follow official guidelines and protocols (in the public sector) versus a more individualised attempt to diagnose the patient prior to the use of tests. For instance, doctors in the public primary care centres make a quick assessment and immediately ask patients to get a sputum test, which is consistent with RNTCP or NTEP guidelines for the public sector in this case.

Our results highlight a fundamental difference between public health rationales and equilibrium patient demand and provider perceptions of that demand, particularly within the comparison between private sector providers and public hospitals. Specifically, the data support the view that patients who report with a 2-week cough and fever in the public sector will immediately be referred for a sputum test. This is the correct protocol-driven response from a public health or technical point of view, as similar ‘rule-based’ triage protocols have been shown to reduce mortality from acute coronary syndrome.^58–60^ However, the likelihood that someone with 2 weeks’ cough in India has TB (without any additional risk factors) is low. Patients may be more motivated to undergo testing if they feel that the healthcare provider is sufficiently attentive to their needs. So, spending more time with the patients and asking more questions and doing basic exams might be helpful in increasing client satisfaction and client retention. This may also attract more people with TB to the public sector.

Since the public sector offers higher-quality TB care from a technical perspective, its free, spare capacity might be filled by demand-side strategies to increase patient demand for public TB services. This capacity is potentially evidenced by the low waiting times in public dispensaries, although not necessarily at higher level facilities like hospitals. One way to do this would be to focus more on patient-centred care in terms of more convenient clinic timings, more time spent with clients, better history-taking and counselling. Recently, MCGM has started to engage TB survivors to improve counselling services once people are diagnosed to have TB. This type of service might improve client satisfaction and retention within the public system. Conversely, given the high volume and revealed patient preferences for care in the private sector, public-private mix interventions should focus on encouraging private providers to notify TB cases, and use microbiological tests such as sputum smears, molecular diagnostics, and DST. This is already being done across India via public-private mix (PPM) programs.

Although this is only the second study to examine public and private sector differences using SPs in India (and the first for urban India), there are several important limitations to the scope of the study. This type of SP study design is not intended to be representative of the average patient experience—it is designed to correctly estimate differences between performance of the average provider in the two sectors for each case presentation only. As a result, it does not account for complex patient pathways, additional potential variation or interaction with patient characteristics like social or demographic variation, accessibility and affordability, complex comorbidities or extended care pathways. For example, if providers first ask for a sputum smear and then ask for Xpert or culture DST subsequently, such sequential interventions will be missed by our cross-sectional design, which only covered first-time SP interactions. Also, the cross-sectional nature of the SP study will not capture the entire patient experience over 6 months of treatment, nor measure quality of treatment support (eg, adherence support), nor capture the costs involved in seeing multiple providers.

The SPs also do not pursue any follow-up care recommended by the providers, so the final standard of care may evolve over follow-up visits with the providers, or change because patients switched providers (including due to referral). Follow-up and referral behaviour have been discussed in other papers with samples and designs appropriate to these questions. These studies show that determining the ultimate quality of care for individual patients is complex and data intensive, but there are two takeaways of importance to this study. First, that single-interaction SPs like those done here are typically predictive of the same provider’s behaviour until the patient initiates a change in course.^25^ Second, referral chains are highly complex in terms of ultimate outcomes and require careful sample selection that we cannot track in this sample.^61^ For this study, our presumption was that any referral is best practice from the perspective of provider behaviour when the provider believes they cannot handle the case themselves.

However, the study accurately highlights the fundamental difference between public and private sector care for TB: a disease and health system where contagion externalities may drive a wedge between what is best for the individual patient and what is best for society as a whole and in which patient choice dynamics and private market incentives may exacerbate this problem.

Overall, our assessment is that both public and private sectors have unique advantages and limitations. While the public sector providers do a good job of adhering to RNTCP or NTEP technical guidelines (eg, microbiological testing) and offer less expensive care, the private sector providers do better on convenience and effort with patients. Public sector doctors were equally well-liked by the SPs; there was no indication that they behaved rudely or inappropriately in any manner; there was no evidence that SPs were asked to pay additional fees beyond the nominal amounts required. The use of medications (including broad-spectrum antibiotics) is equally high in both sectors, and both sectors generally avoided abusing steroids and quinolones.^62^

An interesting implication is that the optimal choice of providers depends on what patients believe. A patient with limited ability to pay who strongly believes that they have TB (or already diagnosed with TB) should go to public sector hospitals (or be referred to the public sector by private and informal providers), where they will receive microbiological TB testing and pay lower out-of-pocket fees, and care that is generally consistent with national guidelines. On the other hand, patients who care about convenience, or who can afford to pay may prefer the private sector. There, more extensive investigations might be done for non-TB illnesses. But this comes at a cost, and the private sector might, at times, order unnecessary tests or treatments.

Given the advantages we found with public sector TB care (ie, lower cost and higher adherence to microbiological testing and NTEP standards), it is important to also work on improving convenience and user experience for patients. This might allow a large fraction of poor TB patients to receive free care in the public sector. To allow poor patients with TB to receive care without catastrophic expenditure in the private sector, public-private partnerships remain important. It is worth noting that Mumbai city has already pioneered a broad-based, successful strategy to engage private providers for TB care via a ‘Private-provider Interface Agency’ (PPIA) model. Through this effort, the programme improved quality of TB care delivered by private providers, including large increases in case notifications, microbiological testing and improved treatment completion, complemented in some cases by cost subsidies.^24^ This model is now being replicated in other parts of India via the Patient Provider Support Agency (PPSA) programme.

## Data Availability

Data are available in a public, open access repository. All questionnaires and case scripts are available from the authors on request. Individual deidentified interaction data, including data dictionaries, will be publicly available online. All variables needed to re-create the results reported in this article will be included, as will the code required to reproduce these results. Data will be available indefinitely on publication to anyone who wishes to access the data for any purpose. The data and code can be accessed and cited at https://doi.org/10.5281/zenodo.4441148.
