# Supplementary material for: Tuberculosis diagnosis and management in the public versus private sector: a standardised patients study in Mumbai, India
Source: BMJ Glob Health. 2022 Oct 19;7(10):e009657. doi: 10.1136/bmjgh-2022-009657 (PMC9582305; doi:10.1136/bmjgh-2022-009657)
Supplement: Supplementary data [file bmjgh-2022-009657supp001.pdf]

Supplemental Appendices for:

Tuberculosis diagnosis and management in public and private health care sectors in Mumbai, India: A cross-sectional standardized patients study

Corresponding author:
Prof Madhukar Pai, MD, PhD
Canada Research Chair in Epidemiology Global Health McGill University
Dept of Epidemiology Biostatistics
1020 Pine Ave West
Montreal, QC H3A 1A2, Canada
Email: madhukar.pai@mcgill.ca

bmjgh-2022-009657 - "Tuberculosis diagnosis and management in public and private health care sectors in Mumbai, India: A cross-sectional standardized patients study"

Appendix – Reflexivity Statement

1. How does this study address local research and policy priorities?

Tuberculosis is a leading killer of people in India, and no country has a higher TB burden than India. Mumbai city has India's biggest burden of drug-resistant TB. So, the study does address a key local and national priority.

2. How were local researchers involved in study design?

The study done in partnership with the Mumbai city government (Municipal Corporation of Greater Mumbai – MCGM), and MCGM partners were involved in the design of the study, and in the interpretation and writing of the results. Six MCGM experts are coauthors of the paper, including DS, the second author. The standardized patient (SP) data collection was also conducted by a team in India (ISERDD).

3. How has funding been used to support the local research team?

The local research team in India was funded to conduct the standardized patient research. Apart from paying the SPs, there were no other major expenses.

4. How are research staff who conducted data collection acknowledged?

All MCGM team members who collaborated as included as co-authors, and the ISERDD team that conducted the SP data collection are acknowledged in the paper.

5. Do all members of the research partnership have access to study data?

All members of the partnership have access to data. The data and code can be accessed and cited at https://doi.org/10.5281/zenodo.4441148.

6. How was data used to develop analytical skills within the partnership?

Data analysis was done by the team at Georgetown university. As such, there was no analytical skill development in this specific project.

7. How have research partners collaborated in interpreting study data?

Study results were shared with the MCGM team and multiple teleconferences were held with their lead (DS).

8. How were research partners supported to develop writing skills?

While there was no plan to refine the writing skills, our partners made extensive edits to various iterations of the manuscript.

9. How will research products be shared to address local needs?

This paper will be published as open access. The MCGM team will share the study results within their TB program and use it for quality improvement of services.

bmjgh-2022-009657 - "Tuberculosis diagnosis and management in public and private health care sectors in Mumbai, India: A cross-sectional standardized patients study"

10. How is the leadership, contribution and ownership of this work by LMIC researchers recognised within the authorship?

Six MCGM experts are coauthors on the manuscript, with DS being the second author.

11. **How have early career researchers across the partnership been included within the authorship team?**

The team includes early career and senior authors, in all the institutions involved.

12. How has gender balance been addressed within the authorship?

Eight authors are female and four authors male

13. How has the project contributed to training of LMIC researchers?

The ISERDD team in India collected the data, and all the SPs were hired as field staff and participated in training and refresher training in order to mitigate any potentially harmful events, such as injections, invasive tests, and consumption of medicines during visits.

14. How has the project contributed to improvements in local infrastructure?

This project has not directly contributed to improvements in local infrastructure.

15. What safeguarding procedures were used to protect local study participants and researchers?

All provider information was treated with confidentiality and not shared in any analysis or report.

Appendix A1

Fieldwork Details

A1.1 Description of SP Case Scenarios

For this study, two tuberculosis (TB) case scenarios were developed to document the level and variation in quality of care for TB among sampled providers. (These two cases have been presented in several of our team's previous publications.) For each case, both the clinical case presentation and social contexts were developed and agreed upon by a technical advisory group, which included clinicians, economists, anthropologists, experts in international and national TB guidelines, and other stakeholders. The two cases were:

1. **Case 1 (Naïve TB Suspect)** – A classic case of presumptive TB with 2-3 weeks of cough and fever. The SP presents to the providers and begins the interaction with the opening statement: "Doctor, I have a cough that is not getting better and some fever too."
2. **Case 2 (MDR Suspect)** – A classic case of presumptive TB with 2-3 weeks cough and fever, and, if asked, a history of previous incomplete TB treatment, which would raise the suspicion of multi-drug-resistant TB.
   (a) **When presenting at Private Facility:** The SP begins the interaction by saying: "Doctor, I am suffering from a bad cough. One year earlier, I had the same – and I was treated at a government hospital – they told me it was TB. I took the medicines then and was better but now the cough has returned."
   (b) **When presenting at Government Facility:** The SP begins the interaction by saying: "Doctor, I am suffering from a bad cough. One year earlier, I had the same – and I was treated by a private practitioner – they told me it was TB. I took the medicines then and was better but now the cough has returned."

A1.2 SP Recruitment, Script Development, and Training

A1.2.1 SP Recruitment

For this study, 10 individuals (3 women) conducted fieldwork between 28/9/2018 and 17/1/2019 in the private sector and between 25/2/2019 and 14/3/2019 for the public sector. For these interactions, 5 SPs (2 women) were Case 1 and 5 SPs (2 women) were Case 4. Some SPs had prior experience as they had participated in our validation study in Delhi (Das et al. 2015) and/or other SP studies assessing quality for other health conditions aside from TB. SPs were originally from

Delhi (1) and the Indian States of Bihar (4) and Maharashtra (5). Primary languages spoken by the SPs included: Hindi (6), Marathi (5), Magahi (2), English (1), and Bangali (1).

During the recruitment process, all potential SPs underwent a health screening questionnaire and checkup, and all SPs in the final cohorts were seemingly healthy, which meant they had no apparent health conditions that could confound the case presentation and interaction with providers. The SPs, although recruited specifically to fit each case scenario and corresponding narrative, differed in age, gender, height, and weight. The average age of all the SPs was 35.5 years. The youngest was 25; the oldest was 51. The 7 men weighed 50 to 61 kilograms and were 162 to 178 centimeters tall. The 3 women weighed 50 to 65 kilograms and were 142 to 160 centimeters tall.

SP Script Development

The following text is an edited version of the Supplemental Appendices from Kwan et al. (2018) and Das et al. (2015), tailored for this study.

Each SP case scenario described above was coupled with a script. Each script is a narrative that describes the social and family contexts of the patient. The scripts were developed under the guidance of an anthropologist (VD) with active supervisor and SP participation. Together, the case scenarios and scripts were piloted in our validation study in Delhi as presented in Das et al. (2015). They were again refined based on field and data management lessons from Delhi and again during and after training in Patna and Mumbai for the study presented in this paper.

The two most important considerations for script development that were also tightly linked to SP training were: First, the clinical symptoms and case history had to reflect the social and cultural milieu of which the SP was assumed to be a member, and second, the presentation of symptoms and answers to history questions had to be consistent with biomedical facts about the disease.

On the former, SPs brought a lot of socially appropriate understanding of the local vocabularies through which symptoms were to be presented and also about typical life histories that would correspond to the age, gender, caste, religion and class of the character that the SP was portraying. As a simple but crucial example, people among the strata the SPs were drawn from do not often use thermometers to measure temperature but report fever on the basis of the sensation of heat and rapid pulse. The inputs by SPs in script development were crucial from this perspective.

The latter issue was to train SPs to present symptoms and answer questions pertaining to case history that were medically correct. For example, all opening statements and questions pertaining to the type of cough and its duration were standardized. A critical part of the training was to help SPs distinguish between questions to which answers could be improvised but had to be appropriate to the social role of the SP and answers that had to be given using local idioms but in a standardized format without any alterations.

The dual aim of presenting the disease in a manner that was not misleading and avoiding detection were largely successful because the reasoning behind both objectives was carefully and repeatedly explained to the SPs and because of their active involvement in the script development and hands-on training. SP case scripts are available from the authors upon request.

A1.2.2 SP Training

The following text is an edited version of the Supplemental Appendices from Kwan et al. (2018) and Das et al. (2015), tailored for this study.

To portray the two SP cases, the individuals recruited as SPs were trained in Mumbai to finalize the case presentation given their knowledge of context, internalize the scripts and cases, be able to

debrief with a supervisor within 1-2 hours of the interaction, and present in clinical settings in a way that would avoid any potentially harmful risks and detection. Thus, SP training was designed with four specific aims:

1. To ensure the SPs correctly present the cases in a standardized way;
2. To ensure the SPs accurately recall the interaction that occurred with health care providers;
3. To ensure SPs avoid both detection or any suspicion that the interaction was not genuine; and
4. To ensure SPs are prepared to avoid potentially harmful risks that can occur to them.

The first two aims were achieved through extensive classroom training in case presentation and recall. Classroom training was complemented with mock interviews and followed by live supervised dry runs in the field at clinics not sampled for the study. Our pilot study in Delhi described in Das et al. (2015) also included the use of tape recorders in a selected subset of interactions, which we used to verify that the results reported on the structured questionnaires were accurate reflections of the clinical interactions.

For the third aim, SPs were carefully instructed to avoid detection by the following methods. First, our recruitment strategy ensured that SPs came from low-income areas or slums from the same cities in which the project was located, so they could easily pass for genuine and local patients, but the areas from which they came were located far from the field sites, so they would not be personally known in the areas they conducted interactions. Second, supervisors for SP fieldwork traveled into the field for 'scoping exercises' before any of the SP interactions were conducted. These scoping exercises helped supervisors to familiarize themselves with landmarks, clinic locations and addresses, general setting, operating hours, length of potential wait time or queues, need for clinic appointments, and other potential issues that could pose challenging to the SP interaction. Based on scoping, the team ensured that SPs were set up to conduct as-successful-as-can-be interactions. Third, during the training, time was organized such that SPs could internalize the characters for each case and the details of their mock stories through which the character was made alive to them. In mock interviews during training, supervisors added unscripted questions with regard to the patient's family or neighborhood details, which SPs were able to answer spontaneously because they were of the actual social background that was being approximated in the characters they were portraying. Finally, dry runs were conducted in which the supervisor was present in the shop on the pretense of buying something, such as toothpaste or an over-the-counter cough syrup, and thus could watch the interaction and use additional training time to improve the SPs' presentations of the cases.

For the fourth aim, SPs participated in active discussions on risk mitigation strategies. Together with the supervisors, the SPs brainstormed what they could do to mitigate risks or avoid situations that could be abnormal. They were then extensively trained on these and additional risk mitigation strategies. (Communication on these matters were prioritized and extended into the data collection period. Throughout the data collection period, which was weathered with scorching heat at times above 43°C/110°F, high humidity, annual monsoons, and flooded roads, supervisor and SP meetings would occur near weekly. These meetings provided additional opportunities to discuss potential issues as a team and how to mitigate them as they were encountered in the field.)

All of these individuals took part in a comprehensive and rigorous 10-day training, which included 3 days of dry runs and was held in Mumbai. The entire standard training was done in Hindi, since even Marathi-speaking individuals were expected to encounter doctors speaking in Hindi, and after classroom training, Marathi-speaking SPs went over scripts and exit questionnaires

in Marathi, followed by dry runs in Marathi. Dry runs were conducted outside of the wards selected for the sample. There were several full meetings for SPs and supervisors, and refresher trainings were conducted every time a new schedule was provided to the field team, approximately every three weeks.

A1.2.3 Rationale for Approved Waiver of Provider Informed Consent

Ethics guidelines on health service audit studies state that SPs should be used in cases where the person being sent the SP is providing a service to other people and where other options have been carefully studied but cannot answer the research questions required. In addition, there should be minimal risk to the participants. Based on 10 years of research, we have demonstrated the difficulties of obtaining quality of care data without using SPs (Alderman et al., 2014). In previous SP studies, we have requested and received waivers of informed consent from ethics committees at Johns Hopkins University, Harvard University, and Duke University. Another SP study conducted at the Universidad Peruana Cayetano Herediahas has also received waiver of informed consent. These waivers have been granted under the provisions for waiver or alteration of the informed consent requirements under the United States Department of Health and Human Services regulations 45 CFR 46.116(d).[1]

Although the ideal study design would include informed consent, we sought a waiver because (a) we were sending multiple SPs to the same healthcare provider, and (b) we would be carrying out these assessments as part of a quality of care surveillance for a TB program (PPIA) being implemented by PATH in Mumbai. In this case, the consequences of potential detection were very high, as the entire 3-year study could have been jeopardized. Further, as part of the PPIA program, providers who were part of the PPIA network were to attend trainings and workshops together. This added a risk to the SP study - for example, if providers in Mumbai were consented for an SP study, the PPIA networked providers could discuss the identities and personal characteristics of the SPs. The combination of informed consent and congregation of providers at frequent intervention trainings (at times several are scheduled in one week) threatened the validity of our study as reported responses would not reflect the actual quality of care we were aiming to measure, while increasing the risk of SP detection.

We therefore worked closely with Institutional Review Board (IRB) requirements on informed consent, which is handled as per the provisions of the Government of Canada Panel on Research Ethics in the 2nd edition of the Tri-Council Policy Statement of Ethical Conduct for Research.[2] Prior to the current study, we conducted a pilot with informed consent to validate the SP method for tuberculosis in urban India (published as Das et al. (2015) in Lancet Infectious Diseases). The results of the pilot validation study confirmed the decision to seek a waiver of informed consent in the current study. Corresponding to the requirements of Article 3.7, we documented in our pilot study that the SP approach in urban India was no more than minimal risk of participation to the SPs or providers. This was based on the following considerations: Involving Humans' Article 3.7 entitled "Alteration of Consent in Minimal Risk Research":

1. Opinion data from providers in the pilot demonstrated that participation in the study did not adversely affect their practice in any way.

[1] Office for Human Research Protections (OHRP). Accessed at: http://www.hhs.gov/ohrp/policy/consentckls.html.

[2] Government of Canada Panel on Research Ethics. 2012. TCPS 2 – Chapter 3, Accessed at: http://www.pre.ethics.gc.ca/eng/policy-politique/initiatives/tcps2-eptc2/chapter3-chapitre3/

2. No monetary loss was incurred by the providers as the SPs, like real patients, paid the full consultation fee.

3. No added inconvenience was placed on real patients as the SPs were trained to immediately step aside if there were an emergency that demanded the doctor's attention.

4. None of the identities of the providers or their clinics were compromised since we maintained strict anonymity in the information collected and never disclosed the identity of health care providers who participated in the study.

5. From our observations, average consultation times in our pilot were between 3 and 7 minutes, so that would only inconvenience other patients by that time.

Based on these considerations, we requested a waiver of informed consent from the IRB at ISERDD and the Research Ethics Board at McGill University. The requests for a waiver were reviewed and approved by both ethics committees, contingent upon the provision of a letter of full disclosure to be sent to debrief any provider who received an SP at the end of the study. The letter will offer health care providers a chance to further discuss any aspect of the findings or methodology and register any concerns; however, no individual data on any clinic or provider will be disclosed. Because our larger quality of care surveillance study has been extended to December 2019, this letter will be circulated thereafter.

A1.3 Sampling and Case Assignment in Private Sector

A1.3.1 Sampling in Private Sector

During the primary data collection period for this study, a Private Provider Interface Agency (PPIA) in Mumbai (led by PATH) was implementing an urban TB program funded by the Bill and Melinda Gates Foundation (BMGF). As the program began, the PPIA were mapping, recruiting, and enrolling private sector providers into provider networks across Mumbai. At the time of sampling for our study, we decided to stratify our provider sample by PPIA program enrollment. From lane-by-lane mapping exercises conducted by the PPIA, which resulted in a universe list of private sector providers in Mumbai, we obtained lists of enrolled and not-enrolled AYUSH practitioners in the PPIA program and merged them to produce a complete sampling universe stratified by PPIA enrollment status.

We then restricted these lists based on eligibility criteria for the SP study: AYUSH practitioners eligible for the study were those who had not yet been enrolled into the PPIA program as of DD MM YYYY and those who had practices in wards of interest for the PPIA expansion at the time. The description of the program serves to support sampling weights (Table 1) applied to achieve the urban area estimates for Mumbai but stratified findings based on PPIA program enrollment are not presented in this paper.

Between January and June 2014 in Mumbai, PATH contracted out street-by-street mapping activities in Mumbai of private health facilities, originally planned for 12 high TB burden wards and 3 high-slum population wards, which account for 86% of the Mumbai slum population and 70% of the Mumbai population. The mapping activity was done by two community-based organizations (Alert India and Maharashtra Janavikas Kendra (MJK)). Mapping was initiated in G-North (high TB burden), M-East (Chembu slums and presence of Chest Physicians), P-North, and S wards. By June 2014, the exercise resulted in a provider universe of 8897 locations in 18 wards, which were 12 fully mapped wards, partial mapping of H-East, P-South, and R-South wards, and minimal

mapping in the remaining wards. Of the 8897, 2804 were chemists, 3591 were AYUSH practitioners (BAMS, BUMS, BHMS, DAMS, DHMS, LCEH), 671 less than fully qualified allopathic providers, 1290 MBBS, 463 MDs, and 78 chest physicians.

Since the network established by PATH aimed to move with the providers regardless of where and in how many facilities they practiced, the final QuTUB sampling frames were the result of thorough cleaning, scoping, and verification exercises in collaboration with the PATH team. Before fieldwork began, an individual (R) hired by ISERDD together with a representative from AHI (NC), both from Mumbai, heavily scoped the field to check for addresses and to gather additional information that would be useful before the ISERDD field team moved to the city. The information that was gathered included, but was not limited to: identifying potential SP recruits, looking at the transportation systems, reporting the setup of clinics and health facilities, collecting GPS locations of providers, and correcting provider and facility names while capturing consulting fees, first points of contact in a facility, and outpatient department (OPD) timings.

Four wards out of the 15 original focus wards of the PPIA program were selected for the majority of Mumbai SP surveillance to minimize geographical spread of the SP study. This was because increasing the geographic scope of the city would increase logistical difficulties as presented by Mumbai's transit system and scale. In mid-January 2015, a meeting was conducted between the PATH and QuTUB teams to agree on the four study wards. After assessing upcoming PPIA program efforts, access to transportation, mixture of unregistered and registered slums, and available data on the wards (e.g., total ward population, slum population, and proportion of slum population figures as per the 2011 census), F-North, K-East, L, and P-North were selected. Relative to other mapped wards, these four wards have a higher slum population (with the exception of F-North), and they also have more providers and more networked providers. Together, these wards were a good representation of areas for PPIA Mumbai efforts. As noted in sections below, these wards were used for constructing the AYUSH sample.

In addition to PPIA hubs, there were non-networked private hospitals and single provider private clinics that met the PPIA hub criteria (i.e., in-house or nearby digital X-ray, pharmacy, and chest physician) and could provide a reasonable comparative 'apples-to-apples' estimate of the quality of major facilities not enrolled in the program. The list of non-PPIA hubs was created with the assistance of PATH, who verified whether each one would be a comparable candidate for enrollment into the program. The list consisted of health facilities that met the criteria for the PPIA network and located in the four study wards: F-North, K-East, L, and P-North wards. An initial list of eligible but non-PPIA 'hubs' was created by PATH in January 2015, and this list was revisited and updated in April 2015. The process of updating the list at the end of April 2015 involved PATH team members and FOs going through the list and removing any facilities that had been networked since July 2015, when SP interactions began for the eligible, yet non-PPIA hubs.

The FOs also went through the full universe list again to make sure the PPIA networking criteria still applied, and any duplicates were removed (at least 12 sets of duplicates or triplicates). The final list was frozen on April 28, 2015, and the QuTUB team flagged facilities that were ineligible for the SP study (i.e., children hospitals) before establishing the sampling frame for non- PPIA hubs. The final list contained 78 eligible, yet non-PPIA hubs, and 11 were excluded from actual SP visits, since they were children or orthopedic hospitals and the SP cases did not reflect pediatric TB or extrapulmonary TB.

Furthermore, we ascertained how PPIA providers (MBBS and higher) practice at PPIA hubs and non-PPIA locations. To expand on this, it was common for individual PPIA providers practicing at PPIA hubs to also have other private practices, which we deem as 'non-PPIA locations' or 'non-networked locations'. These are locations where the providers were confirmed to not be networked regardless of being networked in the PPIA at a different location. To confirm the

PPIA status of these private practices, an initial mapping with verification activities for these 'non-networked locations' occurred between April and July 2015. For initial mapping, two PATH team members in collaboration with the QuTUB team procured a list of practices for 150 PPIA providers, who were MBBS, MDs, and MD/Chest Physicians and who were all networked at known PPIA hubs. Of the 150, 136 met the inclusion criteria (i.e., providers who see adult pulmonary patients) for the SP study.

In terms of the process, FOs conducted the non-networked location mapping in two phases. The first phase, which was circulated to the QuTUB team on July 3rd, contained 153 locations for 62 of the 136 eligible providers and was collected from FOs in R South, N, G North, M East, M West, and F North wards (providers were interviewed at their networked location linked to a ward and a corresponding FO). This resulted in a list of non-networked locations for 62 providers where: 18 providers had 1 location; 21 providers had 2 locations; 10 had 3; 6 had 4; 4 had 5; 2 had 6; and 1 had 7. PATH team members reviewed these locations to determine their networked statuses, and AHI and ISERDD teams conducted scoping for eligibility into the SP study (e.g., government facilities were ineligible for the SP study). The second phase contained details for the remaining 74 providers. For the verification activities, ISERDD and AHI (NC) did on-ground scoping to double-check for any government facilities, ensure there were no duplicates, confirm outpatient hours of the providers, and check whether providers had on-call duties at any of the locations. At the time of verification, ISERDD had already completed all PPIA and non-PPIA hub walk-ins, and favorably, the team was also able to map whether or not any of these non-networked locations had already been visited as a non-PPIA hub. If so, facility IDs matching with those non-PPIA hubs were mapped back to the sample and analytically used also for 'non-networked' interactions.

A1.3.2 SP Case Assignment in Private Sector

Our sampling was restricted to AYUSH practitioners and to four study wards as agreed with PATH. Engagement statuses of AYUSH were frozen on January 24, 2015. There were three samples that resulted: 1. Uniform-probability random sample of non-PPIA AYUSH in K-East and L wards 2. Uniform-probability random sample of non-PPIA AYUSH in F-North and P-North wards 3. Census of PPIA AYUSH in all four wards

Schedules were given to ISERDD in two groups: AYUSH in K-East and L wards in one group, and AYUSH in F-North and P-North wards in a second group. For both groups, reserves were provided for the non-PPIA practitioners based on ward. All AYUSH interactions were randomly assigned to be conducted either in the morning or evening hours.

Next, general walk-ins without appointments to hubs were conducted by having the SP enter the health facility and go to the doctor suggested by the receptionist or intake nurse, without regard for PPIA status of the provider, at both PPIA-registered facilities and non-PPIA facilities. There were two samples: 1. Non-PPIA hub walk-ins at purposively selected locations in the four wards 2. PPIA hubs walk-ins (census of all PPIA hubs) across 15 wards

All hub walk-ins were conducted during specific times that were given to the field team. For PPIA hubs specifically, walk-ins were conducted during hours any networked doctor were scheduled to practice, unless an appointment was scheduled by the receptionist or intake nurse. If, during the consultation, the SP was told to go to another doctor at that moment, the SP was instructed to do so and any aspect of the interaction was also recorded on the same form. On May 1, 2015, before sending SPs to the field, ISERDD supervisors met with the PATH team to go over all the PPIA hubs, their locations, the facility layout, and the networked providers.

Walk-ins were first conducted among 100 PPIA hubs. Only Case 1 interactions were conducted. Once PPIA hub walk-ins were completed, walk-ins were done at non-PPIA hubs. Since we had

to still confirm whether these facilities would accept a presumptive TB walk-in patient, all non-PPIA hubs were sent an SP portraying Case 1. Only after Case 1 interactions were successfully completed, we randomly assigned half to receive Case 2 and then randomly assigned another half to receive either Case 3 or Case 4. None of the SPs trained as Case 4 carried a sputum report to the non-PPIA hubs. To avoid any risk of the same SP running into the same provider across locations, the following field protocols were put in place: (1) SPs were to remain cognizant and maintain a list of providers they had visited, and (2) supervisors were to review a provider directory when assigning SPs for interactions. At least two PPIA providers were seen by SP1 during the non-PPIA walk-in, while 54 of 93 PPIA facility walk-ins resulted in the SP seeing a PPIA provider.

We then targeted additional PPIA providers at facilities where they practiced as part of the PPIA (see Appendix Figure A1). Updated lists for providers, mostly MDs and MD Chest Physicians, networked by the PPIA were procured in January 2015 and then again in April 2015. A total of 136 providers at PPIA hubs were eligible for the SP study (ineligible providers, such as pediatricians and orthopedist specialists, were excluded) across 100 health facilities, resulting in 98 successful interactions. When scheduling the providers and locations that would receive SPs, our top priority was to maximize the total number of interactions we could conduct while reducing risk of detection. In order to do this, we had to work around three issues: (1) providers who were networked at multiple locations, (2) PPIA hubs that had more than 3 networked providers, and (3) providers selected who had already been seen during the walk-in visits. Selecting the eligible providers was done manually and checked several times.

Among the 136 providers, we were aware of 19 who were networked at multiple locations (13 providers at 2 facilities, 2 at 3, 4 at 4 = 48). To maximize the total number of interactions, we then selected these 19 providers at the facility that had the fewest number of other practicing doctors. Among the PPIA hubs, there were six hubs considered as large, defined as having more than 3 networked providers (2 hubs with 5 providers, 1 with 7, 1 with 9, 1 with 10, 1 with 11 = 57). To avoid detection, we decided that no PPIA hub would receive SPs for more than three providers. In order to select the three providers at the large hubs, we attributed the eligible networked doctors to the number of TB cases they had reported to the PPIA program and ranked them from highest to lowest. We then removed all the providers located at multiple locations since we had already selected another one of their networked locations. Three providers were then selected by taking the highest notifying, the lowest notifying, and a random provider who had not been selected yet and who could be either notifying or not to the program.

All PPIA providers at PPIA hubs received SPs portraying Case 1 (however, if SPs as Case 1 had already seen the networked doctor during a walk-in, we did not assign another Case 1 to this doctor, and the assumption was the walk-in observation for Case 1 could be used for the provider targeted interaction for Case 1). A random half received Case 2. Another random half received Case 3 with a sputum report in addition to Case 4 without a sputum report, with the other half was assigned to not receive Case 3 and to receive Case 4 with a sputum report. To minimize detection risk, the field team conducted interactions at the six large facilities last.

Finally, we specifically targeted PPIA providers at their other practice locations where they were not considered part of the PPIA (non-networked locations or "NNLs"). From the verified list of non-networked locations, NNLs that were government facilities and outside Mumbai city were excluded for sampling. Some of the interactions, though not deliberately scheduled for ISERDD, took place during the walk-ins at non-PPIA locations when providers who also practiced within the network were encountered by chance. Scheduling for ISERDD was done in 4 waves as the mapping was done in parallel to fieldwork, and verification process was also done with SP1 case.

PPIA providers at NNLs were scheduled to receive Case 1 before any other cases. This was done to serve as a final verification for location eligibility. Case 1 was randomly assigned to one

Table A1.1: Sampling and SP Case Assignment in Public Sector

**Level**	**Total in Universe**	**Number Sampled**	**Number of Case 1 visits per facility**	**Number of Case 2 visits per facility**	**Total Number of Visits**
Medical colleges	4	4	2	2	16
Peripheral hospitals	16	16	2	2	64
Primary health dispensaries	175	44	1	1	88
Total	195	64			
Total visits			84	84	168

NNL for the providers who SPs visited in the previous sample at a networked location. If the provider was known to have multiple NNLs, we gave preference to the location where he or she was not on-call or working at an actual large PPIA hub before random selection, since large PPIA hubs had already received at least 3 provider-specific SP1s. All on-call locations and other NNLs for each provider was made available as a reserve list to the field team. All PPIA providers at their NNLs were assigned Case 1. The half that did not receive Case 2 at the networked location were assigned to receive them at the NNL. The half that received Case 4 with a sputum report were assigned to receive Case 4 without a sputum report.

A1.4 Sampling and Case Assignment in Public Sector

The public sector sample includes a census of medical colleges managed by MCGM (N = 4), a census of peripheral hospitals managed by MCGM (N = 16), and a random sample of 25% (N = 44 of 175 total) of primary health dispensaries managed by MCGM. This sample was determined by our team in collaboration with MCGM in early 2019 (see Table A1.1). Each medical college or peripheral hospital was assigned two Case 1 and two Case 2 interactions. Each primary health dispensary was assigned one Case 1 and one Case 2 interaction. In all, a total of N = 168 interactions were assigned in the public sector (N = 84 Case 1 visits and N = 84 Case 2 visits; N = 16 in medical colleges, N = 64 in peripheral hospitals, and N = 70 in primary health dispensaries).

A1.5 Medication Coding

After SP interactions were completed with sampled facilities in both cities, a list of all labeled medicines prescribed or offered to the SPs was coded and classified by the team. Medicine received as unlabeled and in loose pills form, such as those received by some AYUSH practitioners in Mumbai, were placed into small plastic bags by the SP field team with one bag for each pill type (defined as same color and shape) and marked as unlabeled on the SP exit questionnaire. The team also determined whether such medicines given included at least one antibiotic or steroid.

In the public sector, medications are given for free as part of the consultation from the facility pharmacy when they are prescribed. In the private sector, practice varies widely. Some providers "bundle" costs; some have attached pharmacies; and some give prescriptions redeemable at outside pharmacies. All costs including medication are included in the statistics reported here. SPs always accepted or purchased medications given during the consultation; in prescription cases, they priced medications at the nearest pharmacy but did not purchase them. When possible, medication costs are individually itemized in the data we provide to accompany the article.

Daniels B, *et al. BMJ Global Health* 2022; 7:e009657. doi: 10.1136/bmjgh-2022-009657

Appendix A2

Standardized Patient Case Scripts

Standardized Case 1: Classic case of suspected TB (with no antibiotics or x-ray)

स्टैन्डराईज्ड केस 1: क्लासिक केस ऑफ सस्पेक्टेड टी बी (बिना एन्टीबायोटिक या एक्स-रे)

स्टैन्डराईज्ड केस 1: क्लासिक केस ऑफ सस्पेक्टेड टी बी (एन्टीबायोटिक या एक्स-रे शिवाय)

Ravi (Male)

Ravi is a 35 year old male who has studied up to 10th standard. He is the owner of a small tea shop. Today, in the morning like any other day, when he leaves for his work, his wife Rekha, handing him his lunch box asks, “why are you not eating your lunch properly - you get most of it uneaten every day”? Ravi replies, “I have cough and seem to have lost my appetite”. Ravi’s family is small. It consists of his wife and two children, aged six (daughter) and four (son) and they live in a two room house which he owns. His business at the tea stall is doing well as he is able to earn on average rupees 12000 - 15000 per month. Generally Ravi keeps good health. He has not had any major health problems or any chronic Illness. His wife and children too are in good health. But since last 2-3 weeks he is suffering from cough which is more or less present during early morning and night, and it also has expectoration though that does not have any color in it and is clear. He also has low grade mild fever, on and off, which gets worse during the evening time. But since this problem started he feels a bit tired and also has lost some weight, as his clothes have got a bit loose. He does not suffer from any associated chest or body pain. He smokes 4-5 *beedis* during the day since last 8-10 years and drinks alcohol once or twice in the month. His relationship with his wife is good. He loves her very much. He has a cheerful and an easy going personality but today his face bears a tense look as he is worried about his cough and fever and visits a doctor nearby.

रवि (पुरुष)

रवि एक 35 साल का व्यक्ति है जिसने 10वीं कक्षा तक पढ़ाई की है। रोजगार के लिये वह अपनी चाय की दुकान चलाता है। जिसे वह खोलने के लिये हर रोज की तरह आज भी अपने घर से निकलता है और निकलते समय उसकी पत्नी रेखा खाने का डिब्बा देते हुये बोलती है कि ''क्या बात है आज–कल आप खाना ठीक से नहीं खा रहे हो''? रवि कहता है ''मुझे खाँसी है और भूख भी कम लग रही है''। रवि का छोटा परिवार है, जिसमें उसकी पत्नी और दो बच्चे है, लड़की की उम्र 6 साल और लड़के की 4 साल है। रवि दो कमरे के खुद के मकान में रहता है। रवि की चाय की दुकान ठीक चलती है जिससे वह औसतन 12 से 15 हजार रुपये महीना कमा लेता है।

आमतौर पर रवि का स्वास्थ्य अच्छा रहता है, उसे किसी भी तरह की तकलीफ और कोई लम्बी बिमारी नहीं है। उसकी पत्नी और बच्चों का स्वास्थ्य भी अच्छा है। लेकिन रवि को पिछले 2–3 हफ्तों से खाँसी है, जो सुबह और रात के समय ज्यादा होती है। उसकी खाँसी के साथ बलगम भी आता है जिसका कोई रंग नहीं है, वह साफ है। खाँसी के साथ उसे हल्का बुखार रहता है जो चढ़ता–उतरता है, लेकिन अक्सर शाम के समय ही ज्यादा होता है। जब से उसे यह तकलीफ शुरु हुई है तब से उसे थकावट महसूस हो रही है। उसे लगता है कि उसका कुछ वजन कम हो गया है क्योंकि उसके कपड़े ढीले हो गये है। उसे इस तकलीफ में किसी भी तरह का छाती का दर्द और बदन दर्द नहीं है।

वह दिन में 4 से 5 बीड़ी पीता है और उसकी यह आदत पिछले 8–10 सालों से है। महीने में एक या दो बार शराब का सेवन भी कर लेता है। उसके अपनी पत्नी के साथ अच्छे सम्बन्ध है। वह उसे बहुत प्यार करता है। वह हंसमुख और मिलनसार स्वभाव का व्यक्ति है, लेकिन आज उसके चेहरे पर अपनी खाँसी और बुखार को लेकर थोड़ी परेशानी है जिसको लेकर वह नजदीक के डॉक्टर के पास गया है।

रवि (पुरुष)

रवि एक ३५ वर्षीय व्यक्ति आहे ज्यांचे शिक्षण १०वी पर्यंत झालेलं आहे. रोजगारासाठी ते स्वतःचा एक चहाचं दुकान चालवतात. आज नेहमीप्रमाणे जेव्हा ते दुकानासाठी निघाले होते तेव्हा त्यांच्या पत्नी रेखा त्यांना जेवणाचा

डब्बा देताना बोलल्या कि “हल्ली असं का होतय की तुम्ही जेवण नीट करत नाही - बहुतांशी अन्न खातच नाही? त्यावर रवि उत्तर देतात कि “मला खोकला झाला आहे आणि भूक पण कमी लागते.” रवि ह्यांचे छोटे कुटुंब आहे, ज्यामध्ये त्यांची पत्नी आणि दोन मुलं आहे, मुलीचे वय ६ वर्ष आणि मुलाचे वय ४ वर्ष आहे. रवि आपल्या परिवारासोबत दोन खोल्यांच्या स्वतःच्या मालकीच्या घरात राहतात. त्यांचे चहाचे दुकान छान चालले आहे ज्याचे उत्पन्न सरासरी १२ ते १५ हजार रुपये दर महिना आहे.

साधारणपणे रवि ह्यांचे आरोग्य चांगले असते, त्यांना कुठल्याही प्रकारचा त्रास किंवा दीर्घकालीन आजार नाही आहे. त्यांच्या पत्नींचे आणि मुलांचे आरोग्य सुद्धा चांगले आहे. रवि ह्यांना मागील २-३ आठवड्यांपासून खोकला सकाळी आणि रात्रीच्या वेळी जास्त होतो. त्यांना खोकल्याबरोबर खाकरा (बेडका) पण येतोय ज्याचा कुठलाही रंग नाही, तो पारदर्शक आहे. खोकल्याबरोबर त्यांना हलकासा ताप चढतो-उतरतो पण अनेकदा संध्याकाळ वेळी ताप जास्त होतो. जेव्हापासून हा त्रास सुरु झाला तेव्हापासून थकवा जाणवत आहे. त्यांना असा वाटतंय कि त्यांचा वजन थोडे कमी झालंय कारण त्यांना कपडे सैल झाले आहेत. त्यांना ह्या त्रासामध्ये कुठल्याहीप्रकारे छाती किंवा अंगदुखी जाणवत नाही.

दिवसातून ते ४-५ बिडी पितात आणि हि सवय त्यांना मागील ८-१० वर्षापासून आहे. महिन्यातून ते एकदा किंवा दोनदा मद्यप्राशन पण करतात. त्यांचे त्यांच्या पत्नीसोबत चांगले संबंध आहेत. त्यांचे त्यांच्या पत्नीवर खूप प्रेम आहे. ते हसतमुख आणि सर्वांशी मिळून मिसळून वागणारे व्यक्ति आहेत, पण आज त्यांच्या चेहऱ्यावर खोकला आणि ताप आल्यामुळे काळजी आहे त्यामुळे ते जवळच्या डॉक्टरांकडे गेले आहेत.

Rekha (female)

Rekha is a 35 year old female who has studied up to 10th standard. She supplements her family income by stitching clothes at home. She is a little worried today as she has cough and running mild fever and thus does not feel like doing work. Her husband suggests that she should go and see a doctor to day about it. He leaves for work at his tea stall. Rekha's is a small family unit with her husband and two children aged six (daughter) and four (son) and they live in a two room house. She has been stitching clothes since a few years as her husband's work does not generate enough income and with her work she is able to earn an extra 4000-5000 rupees in a month. Generally Rekha has been in good health and has not had any major health problems or any chronic Illness. Her husband and children too have had good health. But since last 2-3 weeks she has been having cough which is more or less present during early morning and night, and also has expectoration that does not have any color in it and is clear. She is also running low grade mild fever, on and off, which gets worse during the evening time. But since this problem started she feels tired and also has lost some weight, as her clothes have got bit loose. She does not suffer from any associated chest or body pain. Rekha has a cheerful nature and she abstains from alcohol and smoking. Her relationship with her husband is good and she loves him very much but today she is worried about her cough and fever and visits a doctor nearby

रेखा (महिला)

रेखा पढ़ी–लिखी दसवीं पास 35 साल की महिला है। वह अपने घर के खर्च को पूरा करने के लिये घर में कपड़े सिलाई का काम करती है। आज रेखा का मन काम करने का नहीं कर रहा था क्योंकि आज भी उसे खाँसी, हल्का बुखार और

कमजोरी महसूस हो रही थी। यह बात उसने अपने पति को बताई तो उसके पति ने कहा कि तुम आराम करो और आज किसी डॉक्टर को जरुर दिखा लेना। यह कह कर उसका पति अपनी चाय की दुकान पर चला गया।

रेखा के घर में उनके पति और दो बच्चे है। लड़की की उम्र 6 साल और लड़के की 4 साल है। रेखा दो कमरे के खुद के मकान में रहती है। रेखा कुछ सालों से घर पर सिलाई का काम कर रही है ,क्योंकि उसके पति की चाय की दुकान से इतनी कमाई नहीं हो पाती और वह अपने काम से महीने में 4 से 5 हजार रूपये कमा लेती है।

आमतौर पर रेखा का स्वास्थ्य अच्छा रहता है, उसे किसी भी तरह की तकलीफ और कोई लम्बी बिमारी नहीं है। उसके पति और बच्चों का स्वास्थ्य भी अच्छा है। लेकिन रेखा को पिछले 2–3 हफ्तों से खाँसी है, जो सुबह और रात के समय ज्यादा होती है। उसकी खाँसी के साथ बलगम भी आता है जिसका कोई रंग नहीं है, वह साफ है। उसे हल्का बुखार रहता है जो चढ़ता–उतरता है लेकिन अक्सर शाम के समय ही ज्यादा होता है। जब से उसे यह तकलीफ शुरु हुई है तब से उसे थकावट महसूस हो रही है। उसे लगता है कि उसका कुछ वजन कम हो गया है क्योंकि उसके कपड़े ढीले हो गये है। उसे इस तकलीफ में किसी भी तरह का छाती का दर्द और बदन दर्द नहीं है।

रेखा हंसमुख और मिलनसार स्वभाव की महिला है और वह किसी भी प्रकार का कोई नशा नहीं करती। उसके अपने पति के साथ अच्छे सम्बन्ध है। वह उसे बहुत प्यार करती है। लेकिन आज उसके चेहरे पर अपनी खाँसी और बुखार को लेकर थोड़ी परेशानी है जिसको लेकर वह नजदीक डॉक्टर के पास गयी है।

रेखा (महिला)

रेखा लिहिता-वाचता येणारी दहावी पास ३५ वर्षीय महिला आहे. त्या आपल्या घर-खर्च चालविण्यासाठी घरात शिवण काम करतात. आज रेखांच मन कामावर जाण्यासाठी तयार नव्हतं कारण आज पण त्यांना खोकला, हलकासा ताप आणि अशक्तपणा वाटत होता. हि गोष्ट त्यांनी आपल्या पतींना सांगितली तेव्हा त्यांच्या पतींनी त्यांना आराम करायला सांगितला आणि कोणत्यातरी डॉक्टरांना दाखवायला सांगितले. असे बोलून त्यांचे पती स्वतःच्या चहाच्या दुकानावर निघून गेले.

रेखाच्या घरी त्यांचे पती आणि दोन मुलं आहेत. मुलीचे वय ६ वर्ष आणि मुलाचे वय 4 वर्ष आहे. रेखा दोन खोल्यांच्या स्वतःच्या मालकीच्या घरात राहतात. रेखा काही वर्षापासून शिवण काम करीत आहेत, कारण त्यांच्या पतींच्या चहाच्या दुकानापासून एवढी कमाई होत नाही आणि त्या आपल्या कामापासून महिन्याला ४ ते ५ हजार रुपये कमावतात.

साधारणपणे रेखा ह्यांचे आरोग्य चांगले असते, त्यांना कुठल्याही प्रकारचा त्रास किंवा दीर्घकालीन आजार नाही आहे. त्यांच्या पतींचे आणि मुलांचे आरोग्य   चांगले आहे. पण रेखा ह्यांना मागील २-३ आठवड्यांपासून त्यांना खोकला सकाळी आणि रात्रीच्या वेळी जास्त तीव्र होतो. त्यांना खोकल्याबरोबर खाकरा (बेडका) पण येतोय ज्याचा कुठलाही रंग नाही, तो   पारदर्शक आहे. खोकल्याबरोबर त्यांना हलकासा ताप चढतो-उतरतो   , पण अनेकदा संध्याकाळच्या वेळी ताप जास्त होतो. जेव्हापासून हा त्रास सुरु झाला तेव्हापासून थकवा जाणवत आहे. त्यांना असा वाटतंय कि त्यांचा वजन थोडे कमी झालंय कारण त्यांचे कपडे सैल झाले आहेत. त्यांना ह्या त्रासामध्ये कुठ्ल्याहीप्रकारे छाती किंवा अंगदुखी जाणवत नाही.

रेखा हसतमुख आणि सर्वांशी मिळून मिसळून वागणारे महिला आहेत आणि त्या कुठल्याही प्रकारच्या नशा करीत नाही. त्यांचे त्यांच्या पतिसोबत चांगले संबंध आहेत. त्यांचे त्यांच्या पतिवर खूप प्रेम आहे. पण आज त्यांच्या चेहऱ्यावर खोकला आणि ताप आल्यामुळे काळजी आहे त्यामुळे जवळच्या डॉक्टरांकडे गेल्या आहेत.

Opening statement: **"Doctor, I have cough that is not getting better and some fever too"**

ओपनिंग स्टेटमेंट : डॉक्टर साहब, **"मुझेखाँसी है जो ठीक ही नहीं हो रही है और थोड़ाबुखार भी है"**

ओपनिंग स्टेमेंट : डॉक्टरसाहेब, "मला खोकला झाला आहे जो बरा होत नाहीं आणि थोडा ताप पण आहे"

History questions asked by the provider and their answers

प्रोवाइडर द्वारा पूछे गये हिस्ट्री सवाल और उनके जवाब

प्रोवायडरद्वारा विचारले गेलेले हिस्ट्रीचे प्रश्न आणि त्यांचे उत्तर

Q 1: What is the duration of cough?
प्रश्न 1: खाँसी कब से हो रही है?
प्रश्न 1: खोकला कधीपासून आहे?
Ans 1: 2-3 weeks, more during early morning and night
उत्तर 1: 2–3 सप्ताह से, यह सुबह–सुबह और रात को ही ज्यादा होती है।
उत्तर 1: २-३ आठवड्यांपासून, सकाळी-सकाळी आणि रात्री जास्त होतो

Q2: Are you producing sputum (bulgam)?
प्रश्न 2: क्या आपको बलगम बनती है?
प्रश्न 2: खाकरा/बेडका/थुंकी मध्ये कफ तयार होतो का?
Ans 2: Yes
उत्तर 2: हाँ।
उत्तर 2 : हो

Q3: Does the sputum contain blood?
प्रश्न 3: क्या आपके बलगम में खून आता है?
प्रश्न 3. खाकरा/बेडका/थुंकी मध्ये रक्त पडते का?
Ans 3: No
उत्तर 3: नहीं।
उत्तर 3: नाही

Q 4: How long have you had fever?
प्रश्न4: आपको बुखार कब से है?
प्रश्न 4. आपल्याला ताप कधीपासून आहे?

Ans 4: Since 2-3 weeks
उत्तर 4: 2–3 हफ्ते हो गये।
उत्तर 4 : २-३ आठवड्यांपासून

Q5: What type of fever do you have?
प्रश्न5: बुखार कैसा रहता है?
प्रश्न 5 : ताप कसा असतो?

Ans 5: Low grade (mild), on and off, more during evening times.
उत्तर 5: हल्का बुखार चढ़ता उतरता रहता है, लेकिन ज्यादातर शाम को होता है।
उत्तर 5. हलकासा ताप चढतो-उतरतो, पण जास्तीकरून संध्याकाळी वाढतो

Q 6: Have you taken any medicines for your illness?
प्रश्न 6: क्या आपने इस तकलीफ के लिये कोई दवाई ली है?
प्रश्न 6. ह्या आजारासाठी आपण कोणती औषधे घेतली आहेत का?

Ans 6: No.
उत्तर 6: नहीं।
उत्तर 6: नाही

Q7: Do you get any chest pain?
प्रश्न7: क्या आपकी छाती में दर्द होता है?
प्रश्न 7: आपल्या छाती मध्ये दुखतंय का?

Ans 7: No
उत्तर 7: नहीं।
उत्तर 7: नाही

Q8: Any loss of appetite?
प्रश्न8: भूख में कोई कमी?
प्रश्न 8: भूक कमी झाली आहे का?

Ans 8: Yes, loss of appetite.
उत्तर 8: हाँ, भूख तो कम लगती है।
उत्तर 8. हो, भूक कमी लागते.

Q9: Have you lost weight?
प्रश्न9: क्या वजन कम हुआ है?
प्रश्न 9: आपलं वजन कमी झाले आहे का?

Ans 9: I think so; my clothes have become a bit loose.
उत्तर 9: हाँ, मुझे लगता है क्योंकि मेरे कपड़े ढीले हो गये हैं।
उत्तर 9 : हो, मला वाटतंय कारण माझे कपडे सैल होत आहेत

Q10: Any wheezing or difficulty in breathing?

प्रश्न10: साँस लेने में कोई तकलीफ/सीटी जैसी आवाज?

प्रश्न 10 : श्वास घेताना/सोडताना कुठली अडचण होते का/छाती मधून घरघर (शिटी सारखा) आवाज येतो का?

Ans 10: No

उत्तर 10: नहीं।

उत्तर 10: नाही

Q11: Do you smoke?

प्रश्न11: क्या आप बीड़ी/सिगरेट पीते हैं?

प्रश्न11: आपण विडी-सिगारेट पिता का?

Ans 11: Yes, I smoke beedis. [in case of male SP]

उत्तर 11: हाँ, मैं बीड़ी पीता हूँ। (मेलSP के लिये)

No [in case of females SP] नहीं (फीमेल SP के लिये)

उत्तर ११: हो, मी बीडी/सिगारेट (पुरुष एस पी साठी) पितो, नाही (स्त्री एस पी साठी)

Q 12: How manybeedis in a day?

प्रश्न 12: एक दिन में कितनी पीते हो?

प्रश्न 12: एका दिवसात किती बीडी/सिगारेट पिता?

Ans 12: 4-5beedis, I guess

उत्तर 12: अंदाजन, 4 से 5 बीड़ी।

उत्तर 12 : अंदाजे, ४ ते ५ बीडी/सिगारेट

Q 13: Since when have you been smoking beedis?

प्रश्न 13: कब से बीड़ी पी रहे हो?

प्रश्न 13:आपण कधीपासून बीडी/सिगारेट पीत आहात?

Ans 13: Since the last 8 or 10 years

उत्तर 13: पिछले 8 या 10 सालों से।

उत्तर 13: मागील ८ ते १० वर्षांपासून

Q 14: Do you drink alcohol?

प्रश्न 14: क्या आप शराब पीते ेऽæ

प्रश्न 14: आपण दारू पिता का?

Ans 14: Yes [in case of male] No [in case of female SP]

उत्तर 14: हाँ (मेल SP के लिये), नहीं (फीमेल SP के लिये)

उत्तर 14: हो, मी बीडी/सिगारेट (पुरुष एस पी साठी) पितो, नाही (स्त्री एस पी साठी)

Q 15: How often do you drink?

प्रश्न 15: कितनी बार पी लेते हो?

प्रश्न 15: आपण कितीवेळा दारू पिता?

Ans 15: Once or twice in a month.[in case of male] No [in case of female SP]

उत्तर 15: महीने में एक–दो बार। (मेलSP के लिये), नहीं (फीमेल SP के लिये)

उत्तर 15: महिन्यातून एकदा-दोनदा (पुरुष एस पी साठी) , नाही (स्त्री एस पी साठी)

Q 16: Have you been treated for TB in the past?

प्रश्न 16: क्या आपने पहले कभी टी बी का इलाज कराया है?

प्रश्न 16: आपण पूर्वी कधी टी बी साठी उपचार घेतले होते का?

Ans 16: No

उत्तर 16: नहीं तो।

उत्तर 16: नाही

Q 17: Anyone in your family has TB?

प्रश्न 17: क्या आपके घर में किसी को टीबी है?

प्रश्न 17: आपल्या घरी कोणाला टी बी आहे का?

Ans 17: No

उत्तर17: नहीं।

उत्तर17: नाही

Q 18: Do you have diabetes?

प्रश्न 18: क्या आपको शुगर ≥æ

प्रश्न 18: आपल्याला साखरेचा त्रास आहे का?

Ans 18: I do not know

उत्तर 18: जी,पता नहीं।

उत्तर 18: माहित नाही

Q 19: Do you have hypertension?

प्रश्न 19: क्या आपको हाईपरटेंशन है?

प्रश्न 19: आपल्याला हायपरटेंशन/रक्तदाबाचा त्रास आहे का?

Ans 19: I do not know

उत्तर 19: जी,पता नहीं।

उत्तर 19: माहित नाही

Q20: Do you have HIV-AIDS?

प्रश्न20: क्या आपको HIV-AIDS है?

प्रश्न20: आपल्याला HIV-AIDS आहे का?

Ans 20:I do not know

उत्तर 20: जी,पता नहीं।

उत्तर 20 माहित नाही

Q21: Have you ever been tested for these diseases?
प्रश्न21: क्या आपने कभी इन बिमारियों कि जाँच या टेस्ट करवाया है?
प्रश्न21: काय आपण ह्या रोगांसाठी तपासण्या केल्या आहेत का?
Ans21: Not been tested
उत्तर 21: कभी टेस्ट नहीं करवाया।
उत्तर 21: कधी तपासण्या नाही केल्या

Q 22: Are you allergic to any drugs?
प्रश्न 22: क्या आपको किसी दवाई से एलर्जी है?
प्रश्न 22. आपल्याला कुठल्याही औषधापासून एलर्जी आहे का?
Ans22: No.
उत्तर 22: नहीं।
उत्तर 22: नाही

Important instructions to be remembered by SP

महत्वपूर्ण बातें जो एस पी को याद रखनी है

एस पी साठी महत्वाच्या सूचना

1.	ISERDD staff will identify the facility and/or the provider for the SP, where the SP will present his case alone.<br>ISERDDस्टाफ फील्ड मेंSPको प्रोवाइडर का क्लीनिक बतायेगा जहाँSPको अकेले अपना केस करना है।<br>ISERDD स्टाफ फील्ड मध्ये SP ला प्रोवायडरचे क्लिनिक सांगितले जातील, तिकडे SP ला एकटेच आपली केस सादर करायची आहे।
2.	SP should remember if the facility and/or the provider was the correct one. (for example, it was the correct location, but the name board did not match the name given or if the assigned provider was not there and the SP saw another provider)<br>SPको याद रखना है कि वो सही प्रोवाइडर के क्लीनिक गये थे? (उदाहरण के लिए, जगह सही थी लेकिन बोर्ड पर लिखा नाम दिये गये नाम से मैच नहीं कर रहा था।<br>SPनी लक्षात ठेवायचे आहे कि ते योग्य प्रोवायडरच्या क्लिनीक मध्ये गेले होते का? (उदाहरणासाठी जागा बरोबर होती पण बोर्डावरचे नाव दिलेल्या नावाशी जुळत नव्हते)
3.	SP must remember if the provider carried out any of the following examination?<br>SP को याद रखना है कि प्रोवाइडर ने निम्नलिखित में से कोई परीक्षण किये?<br>SPनी लक्षात ठेवायचे आहे कि प्रोवायडरनी खालीलपैकी कोणत्या तपासण्या केल्या?<br>• Pulse rate नब्ज़ की दर नाडीचे स्पंदन मोजणे<br>• Respiratory rate साँस की दर श्वसन दर मोजणे

	• Auscultation of Chest(Did the provider comment on the auscultation)<br>हृदय, फेफड़ों की गति को सुनना (क्या प्रोवाइडर ने ऑस्कलटेशन करते समय कुछ कहा जैसे आवाज साफ है या अन्य)<br>हृदय फुफ्फुसे अंतर्गत होणाऱ्या आवाज ऐकून तपासणे (प्रोवायडर ने ऑस्कलटेशन करताना काय सांगितलं का जसे आवाज साफ आहे किंवा अन्य)<br>• Blood Pressure ब्लड प्रेशर ब्लड प्रेशर / रक्तदाब मोजणे<br>• Temperature बुखार मापना – थर्मामीटर ताप मापन - थर्मामीटरने<br>• Throat examination गले का परीक्षण गळ्याचे परीक्षण<br>• Weight वजन मापना वजन मापन<br>• Abdomen palpitation पेट दबाकर परीक्षण पोटाचे दाबून परीक्षण करणे
4.	Did the provider recommend any investigations? क्या प्रोवाइडर ने निम्नलिखित जाँच कराने को कहा?<br>प्रोवायडरनी खालीलपैकी कोणत्या तपासण्या करायला सांगितल्या?<br>(If yes, SP should ask provider to write the name of the test and the laboratory. And hand over the document to an ISERDD staff.) *Write the specific name of lab given by the provider and if no put (-99).*<br>(यदि हाँ, तो SPको प्रोवाइडर से टेस्ट और लैब का नाम लिखित में लेना है और उस पर्चे को ISERDD स्टॉफ को सौंप देना है) *अगर प्रोवाइडर ने किसी विशेष लैब का नाम दिया है तो वह लिखें, यदि नहीं तो (-99) भरें।*<br>(जर हो तर, SP नी प्रोवायडरकडून टेस्ट आणि लैबचे नाव लिहून घ्यायचे आहे आणि त्या कागदाला ISERDD स्टाफला द्यायचे आहे) जर प्रोवायडरनी विशेष लैब चे नाव सुचवले तर ते लिहून घ्या, जर नाही सांगितले तर (-99) भरा<br>• Chest X-Ray छाती का एक्स-रे छातीचा एक्स-रे<br>• CT Scan सी टी स्केन सी टी स्कॅन<br>• Blood- Total count, differential count- ESR रक्त–टोटल काउंट, डिफरेंशियल काउंट–ई एस आर<br>रक्त टोटल काउंट डिफरेंशियल काउंट - ई एस आर<br>• Blood- HIV test रक्त–एच आईवी टेस्ट रक्त एच आय वी टेस्ट -<br>• Blood- Diabetes test रक्त–शुगर टेस्ट रक्त शुगर टेस्ट -<br>• Blood- TB Gold रक्त – टी बी गोल्ड रक्त टी बी गोल्ड टेस्ट -<br>• Blood-TB ELISA रक्त – टी बी ऐलाइजा रक्त टी बी ऐलाइजा -<br>• Sputum smear examination (Sputum AFB) स्पूटम स्मियर एक्जामिनेशन<br>स्पूटम स्मियर एक्जामिनेशन स्पूटम ए एफ बी /<br>• Sputum GeneXpert test स्पूटम जीनएक्सपर्ट टेस्ट स्पूटम जीनएक्सपर्ट टेस्ट<br>• Sputum culture स्पूटम कल्चर स्पूटम कल्चर<br>• Mantoux Test मॉनटुक्स टेस्ट मौनटूक्स टेस्ट<br>• TB- PCR (not specific) टी बी – पी सी आर (विशिष्ट नहीं है) टी बी - पी सी आर (विशेष नाही आहे)<br>• Drug susceptibility test (culture, gene Xpert, line probe assay) ड्रग स्सेप्टिबिलिटी टेस्ट (कल्चर, जीनएक्सपर्ट, लाइन प्रोव एस्से) ड्रग स्सेप्टिबिलिटी टेस्ट (कल्चर, जीनएक्सपर्ट लाईन प्रोब एस्से)

5.	Did the provider give you any vouchers or referral coupons?<br>क्या प्रोवाइडर ने SP को कोई वाऊचर या रेफरल कूपन दिया? प्रोवायडरनी SPला कुठले वाऊचर/रेफेरल कूपन दिले का?<br>(If yes, SP should retain the voucher or referral coupons and hand over to any ISERDD staff. On the form, supervisors will write the voucher number and name from the stamp.)<br>(यदि हाँ, तो SP को वाऊचरया रेफरल कूपन सभांल कर रखना है और ISERDDस्टाफ के हाथों में सौंपना है)<br>(जर हो, तर SP नी वाऊचर/रेफेरल कूपन सांभाळून ठेवायचे आहे आणि ISERDD स्टाफ च्या हातात द्यायचे आहे)<br>• Treatment उपचार उपचार<br>• Chest X-Rayछाती का एक्सरे छातीचा एक्स-रे<br>• GeneXpertजीनएक्सपर्ट टेस्ट जीनएक्सपर्ट टेस्ट<br>Please write voucher or referral coupons number and name on the stamp.<br>कृप्या वाऊचर या रेफरल कूपन का नम्बर तथा स्टैम्प पर जो नाम लिखा है उसे लिखें।<br>कृपया वाऊचर किंवा रेफरल कूपन चा नंबर आणि स्टैम्पवर जे नाव लिहिलेलं असेल ते लिहा
6.	SP must remember if personal details (Name, Mobile, Address) were given to the provider. If so, what was the full name the SP gave? (Some providers who work with the PPIA will collect this information if they identify the SP as a presumptive TB case, refer you to a lab for sputum or CXR, or refer you to certain providers.)<br>SPको याद रखना है कि उसनेप्रोवाइडर को क्या निजी जानकारी दी। जैसे नाम, मोबाइल नम्बर, पता। यदि दी, तो एस पी ने अपना पूरा नाम क्या बताया? (PPIA के साथ काम करने वाले कुछ प्रोवाइडरइस जानकारी को इकट्ठा करेंगे यदि वह पहचान ले कि आपएक संभावितTBकेस हो तो वह आपको छाती एक्स–रे या थूक जाँच के लिए लैब में रेफर या चुने हुए प्रोवाइडर के पास रेफर करेंगें)<br>SPनी लक्षात ठेवायचे आहे कि त्यांनी कोणती वैयक्तिक माहिती प्रोवायडरला दिली आहे. जसे आपले नाव, मोबाईल नंबर, पत्ता, जर दिला असेल, तर SPनी आपलं पूर्ण नाव काय सांगितलं? (PPIA च्या बरोबर काम करणारे काही प्रोवायडरर्स आपली माहिती एकत्रित करतील आणि जर त्यांना वाटलं कि आपण एक संभावित TB केस आहात तर ते आपल्याला छातीचा एक्स-रे किंवा थुंकीची तपासणीसाठी लैब मध्ये रेफर किंवा निवडलेले प्रोवायडरकडे जाण्यासाठी रेफर करतील)
7.	SP must remember if someone at the facility completed a registration form to register the SP's personal details such as with a call center. (Some providers who work with the PPIA will make a call to register patients.)<br>SP को याद रखना है कि प्रोवाइडर ने SP की व्यक्तिगत जानकारी रजिस्टर करने के लिए फोन एक कॉल सेंन्टर को फोन किया।<br>(PPIA के साथ काम करने वाले कुछ प्रोवाइडर पेशेंट को रजिस्टर करने के लिए फोन करेंगे।)<br>(SPनी लक्षात ठेवायचे आहे कि जर कोणी फैसिलीटीमध्ये कोणी SPची वयतिक्त माहितीची नोंदणी करण्यासाठी कॉलसेंटरला कळविले (काही प्रोवायडर जे PPIA बरोबर काम करतात ते पेशंट रजिस्टर करण्यासाठी कॉल करतील)
8.	ISERDD staff to mark which of the following tests was recommended by a networked provider.(Refer to networked provider/lab list)<br>जो भी टेस्ट नेटवर्क प्रोवाइडर द्वारा करवाने के लिए बोला गया है उसेISERDD स्टॉफ को फार्म में मार्क करना है।(नेटवर्क प्रोवाइडर/लैब पर रेफर किया)<br>जी कोणतीही टेस्ट नेटवर्क प्रोवायडरद्वारा करण्यातही सांगितली आहे ती टेस्ट ISERDD स्टाफने फॉर्ममध्ये

	मार्क करायची आहे (नेटवर्क प्रोवायडर / लैब ला रेफर केलं)
9.	SP must collect prescription and/or any medicines given by the provider.<br>SPको प्रोवाइडर द्वारा दी गयी दवाई या दवाई का पर्चा अवश्य लेना है।<br>SPनी प्रोवायडरने दिलेली औषधं आणि औषधांच कागद घ्यायचा आवश्यकचं आहे
10.	SP must remember if the provider gave any diagnosis.<br>SPको प्रोवाइडर द्वारा दिये गये डाइग्नोसिस को याद रखना है।<br>SPनी प्रोवायडरने दिलेल्या डायग्नोसिस ला लक्षात ठेवायचे आहे?
11.	SP must remember if the provider recorded the information he took from you.<br>SPको यह ध्यान रखना है कि प्रोवाइडर ने आपसे जो जानकारी ली उसको कहीं लिखकर रखा।<br>SPने लक्षात ठेवायचे आहे कि प्रोवायडरला आपण दिलेली माहिती त्यांनी कुठे लिहून नोंदली का?:
12.	SP should remember if the provider referred to: SPको याद रखना है यदि प्रोवाइडर रेफर करता है तो :<br>जर प्रोवायडरने रेफर केलं तर SPनी खालील गोष्टी लक्षात ठेवायचे आहेत<br>• a private provider/ private hospitalप्राईवेट प्रोवाइडर/प्राईवेट अस्पताल प्रायवेट प्रोवायडर / प्रायवेट रुग्णालय<br>• BMC / Municipal hospital बी एम सी / म्युनिसिपल अस्पताल बी एम सी / म्युनिसिपल रुग्णालय<br>• DOTS centreडॉट्स सेन्टर डॉट्स सेंटर<br>• Other locationअन्य स्थान अन्य जागा<br>and include the name of the provider or health facility you were referred to:<br>जहाँ रैफर किया है उस प्रोवाइडर या हेल्थ फैसिलिटी का नाम शामिल करे :<br>आणि जिकडे रेफर केलं त्या प्रोवायडर किंवा फैसिलीटीचे नाव सामील करा
13.	Prescriptions and pills given must be preserved for analysis.<br>SPको पर्चा और दवाईयाँ एनालिसिस के लिये संभाल कर रखना है।<br>SPनी डॉक्टरांनी दिलेला कागद आणि औषधं-गोळ्या एनालीसिससाठी सांभाळून ठेवायच्या आहेत
14.	SP should refuse any injections/ invasive tests performed by the provider during this encounter but note down details of what was offered/suggested.<br>SPको इस बातचीत के दौरान किसी भी तरह का इन्जेक्शन/इन्वेसिव टेस्ट लेने से इन्कार करना है लेकिन ऐसे किसी भी सुझाव को नोट करके बताना है।<br>SP नी संभाषण दरम्यान कुठल्याही प्रकारचे इंजेक्शन / इन्वेसिव टेस्ट घ्यायला नकार द्यायचा आहे आणि अश्या प्रकारच्या सूचना नोट करून ठेवायच्या आहेत
15.	If the provider asks any question that is not in the list then the SP should say no or deflect by either saying I don't know or I don't remember as is appropriate to the context.<br>यदि प्रोवाइडर कोई ऐसा सवाल पूछता है जो लिस्ट में शामिल नहीं है तो SP को कहना है मुझे पता नहीं या याद नहीं आ रहा (जो उस समय उचित लगे)<br>जर प्रोवायडर आपल्याला असा प्रश्न विचारतात जो लिस्ट मध्ये सामील नाही आहे तर SP नी सांगायचे आहे कि मला माहित नाही किंवा आठवत नाही (जे त्या वेळी उचित वाटेल ते)
16.	SP should get the prices of the prescribed medicines, labs or consultation fees from the provider.<br>SP को प्रोवाइडर द्वारा पर्चे पर लिखी दवाई, लैब या कन्सल्टेशन फीस की जानकरी को याद रखना है।

	SPनी प्रोवायडरद्वारा दिलेला कागदावर लिहलेली औषधं, लैब किंवा कन्सलटेशन फीज ची माहितीला लक्षात ठेवायचे आहे
17.	Supervisor /SP should get the prices, brand and generic names of the prescribed medicines.<br>Supervisor/SP को प्रोवाइडर द्वारा लिखी दवाई का मूल्य, ब्रांड तथा जेनरिक नाम का पता करना है।<br>Supervisor/ SP ला प्रोवायडरद्वारा दिले गेलेल्या औषधांचे मूल्य, ब्रांड आणि जेनरिक नावांची माहिती गोळा करायची आहे<br>• Fill all unlabelled FIRST खुली दवाईयाँ पहले लिखें खुली औषधं पहिले लिहा<br>• In brand: write Unlabelled ब्रॉण्ड में अनलेबल्ड लिखें ब्रांड मध्ये अनलेबल्ड लिहा<br>• In generic: describe the color + shape जेनेरिक में दवाई का आकार व रंग लिखें जेनेरीक मध्ये रंग आणि आकार लिहा

Standardized Case 4: A case of suspected MDR-TB (previous history of TB treatment)

स्टैंडर्ड केस 4ः एम आर डी – टी बी (पहले टी बी का इलाज़ लिया हुआ है)

स्टैन्डराईज्ड केस 4: एम आर डी – टी बी (ह्या पूर्वी टी बी चा उपचार घेतला आहे)

Suraj (Male)

Suraj is a 38 year old male who has studied upto 10th standard while he was living with his uncle (Chacha) in the city. He works in a General Store and earns rupees 14000 to 15000 in a month. He lives in the city in a rented two room house with his wife and three children, aged 13 (boy), 10 (girl) and 6(boy) and all of them are studying in a BMC / Municipal school. His parents are back in the village where his father has a small piece of land.

Suraj had developed a cough last year for which he went to a BMC / Municipal hospital near his house where after a sputum test and chest x-ray he was diagnosed with TB. He was given 6 or7 different types of tablets and had to take them on alternate days. (ek din chod kar). He took the treatment for 4 to 5 months and then stopped it as he felt better.But after stopping the medication he had mild cough which used to subside on its own. His family has been keeping well and none of them have ever had any of hissymptoms.

But since last one month he is suffering from cough which is more or less present during early morning and night, and he also has expectoration in which he has seen flecks of blood once or twice in a month. He also has low grade mild fever, on and off, which gets worse during the evening time. But since this problem started he feels a bit tired and also has lost some weight, as his clothes have gotten a bit loose. He does not suffer from any associated chest or body pain. He has tried some home remedies and cough syrup from a local chemist for the problem.However, the cough had not subsided.He has misplaced other medical records.
He smokes 4-5 *beedis* during the day since last 10-12 years and drinks alcohol once or twice the month.

सूरज (पुरुष)

सूरज 38 साल का व्यक्ति है जिसने शहर में अपने चाचा के पास रहकर 10वीं कक्षा तक पढ़ाई की है। सूरज के माता–पिता के पास गाँव में खेती के लिए छोटी सी जमीन है। सूरज अपनी पत्नी और तीन बच्चों के साथ जिनकी उम्र 13 (लड़का), 10 (लड़की) और 6 (लड़के)साल की है जो किराये के दो कमरों के मकान में रहते है। सूरजएक किराना की दुकान (जनरल स्टोर) में काम करता है जहाँ उसेमहीने के 14000 से 15000 रूपये तनख्वाह मिलती है। उसकेतीनों बच्चे सरकारी स्कूल में पढ़ते है।

सूरज को पिछले साल खाँसी हो गयी थी जिसके लिये उसने घर के पास ही एक **बी एम सी / म्युनिसिपल अस्पताल** अस्पताल से इलाज कराया था। वहाँ थूक और एक्स–रे की जाँच के बाद पता चला की उसे टी बी है।वहाँ से 6–7 तरह की दवाईयाँ मिलती थी, जिन्हे एक दिन छोड़कर खाना होता था। उसने यह इलाज 4–5 महीनेलिया और फिर बन्द कर दियाक्योंकि उसे लगा कि उसे आरामआ गया है। इलाज छोड़ने के बाद सूरज को बीच–बीच में खाँसी हो जाती थी मगर वह अपने आप ही ठीक हो जाती थी। सूरज की पत्नी और बच्चों का स्वास्थ्य अच्छा है और किसी को भी कोई लम्बी बिमारी नही रही।

लेकिनपिछले एक महीने से सूरज को फिर से खाँसी और बुखार की परेशानी हो रही है।खाँसी के साथ बलगम भी आता है जिसमें पिछले महिने में एक–दो बार लाल रंग के धब्बे भी दिखाई दिये है।उसे हल्का बुखार है, जो चढ़ता–उतरता रहता है पर अक्सर शाम के समय ही ज्यादा रहता है। जब से उसे यह तकलीफ शुरु हुई है तब से उसे थकावट महसूस हो रही है। उसे लगता है कि उसका कुछ वजन कम हो गया है क्योंकि उसके कपड़े ढीले हो गये है। उसको किसी भी तरह का

छाती और बदन दर्द नहीं है। सूरज ने पिछले महीने कैमिस्ट से खाँसी का सिरपऔर कुछ घरेलू उपचार किये जिससे उसकी खाँसी में कुछ आराम नहीं आया। लेकिन उसे लग रहा है कि अस्पताल वाले उसे इधर से उधर चक्कर लगवायेंगे, इसलिए उसने प्राइवेट डॉक्टर से सलाह लेने के बारे में सोचा। उसके पास थूक की जाँच की रिपोर्ट है लेकिन बाकी मेडिकल रिकार्ड उससे कहीं खो गये है।

सूरज दिन में 4 से 5 बीड़ी पीता है और उसकी यह आदत पिछले 10–12 सालों से है। महिने में एक या दो बार शराब का सेवन भी कर लेता है।

सूरज (पुरुष)

सूरज एक ३८ वर्षीय व्यक्ति आहेत ज्यांनी आपल्या काकांकडे राहून १०वी पर्यंतचं शिक्षण पूर्ण केलं. सुरज ह्यांच्या आई-वडिलांकडे गावामध्ये शेतीसाठी एक छोटीशी जमीन आहे. सुरज आपल्या पत्नी आणि तीन मुलांसोबत, ज्यांची वयं १३ (मुलगा), १० (मुलगी) आणि ६ (मुलगा) आहेत, भाड्याच्या दोन खोल्यांच्या घरात राहतात. सुरज एका किराणा दुकान (जनरल स्टोर) मध्ये काम करत आहेत ज्या मध्ये त्यांना प्रति महिना 14००० ते १५००० रुपये पगार मिळतो. त्यांची तिन्ही मुलं बी एम सी / म्युनिसिपल शाळेमध्ये शिकत आहेत.

सुरज ह्यांना मागील वर्षी खोकला झाला होता ज्यासाठी त्यांनी त्यांच्या घराजवळच्या बी एम सी / म्युनिसिपल रुग्णालयात उपचार घेतले होते. तिकडे थुंकी आणि एक्स-रे च्या तपासणी केल्यानंतर कळलं कि त्यांना टी बी आहे. तिकडून ६-७ प्रकारच्या औषध-गोळ्या मिळत होत्या, ज्यांना एक दिवस सोडून घ्यायचं असायचं. त्यांनी हा उपचार ४-५ महिने घेतला आणि नंतर बंद केला कारण त्यांना बरं (आराम) वाटू लागले होते. उपचार बंद केल्यानंतर सुरज ह्यांना अधून-मधून खोकला येत असे पण तो आपोआपच ठीक होत होता. सुरज ह्यांच्या पत्नी आणि मुलांचे आरोग्य चांगले आहे आणि कोणालाही दीर्घकालीन आजार नाही आहे.

पण मागील एका महिन्यापासून सुरज ह्यांना परत खोकला आणि तापाचा त्रास होऊ लागला आहे. खोकल्याबरोबर खाकरा (बेडका) पण येतोय ज्यामध्ये मागील महिन्यात एक-दोन वेळा लाल रंगाचे धब्बे पण दिसले होते.खोकल्याबरोबर त्यांना हलकासा ताप चढतो-उतरतो, पण अनेकदा संध्याकाळच्या वेळी ताप जास्ती होतो. जेव्हापासून हा त्रास सुरु झाला तेव्हापासून थकवा जाणवत आहे. त्यांना असा वाटतंय कि त्यांचा वजन थोडे कमी झालंय कारण त्यांना त्यांचे कपडे सैल झाले आहेत. त्यांना ह्या त्रासामध्ये कुठल्याहीप्रकारे छाती किंवा अंगदुखी नाही झाली आहे.सुरजने मागील महिन्यात केमिस्टकडून खोकल्यासाठी सिरप अणि काही घरगुती उपचार केले तरीसुद्धा त्यांच्या खोकल्याला काही आराम मिळाले नाही. त्यांना असा वाटतंय की रुग्णालयात त्यांना इकडून तिकडे चक्कर मारायला लावतील म्हणून त्यांनी प्रायवेट डॉक्टरांचा सल्ला घ्यायचा विचार केला आहे. त्यांच्याकडे थुंकीच्या तपासणीचा रिपोर्ट आहे पण बाकिचे मेडीकल त्यांच्याकडूनच रेकॉर्ड हरवले आहेत.

सूरज दिवसातून ४ ते ५ बीड़ी पितात आणि त्यांची ही सवय मागील १०-१२ वर्षांपासूनची आहे. ते महिन्यातून एकदा किंवा दोनदा मद्यप्राशन पण करतात.

Sapna (Female)

Sapna is a 38 year old female, a home maker, who has studied upto 10th standard. Her husband works in a General Store and earns rupees 15000 in a month. She lives in a rented two room housewith her husband and three childrenaged 13 (boy), 10 (girl) and 6(boy). Her two elder children are studying in a BMC / Municipal school and the youngest is attending a small play school nearby. She is able to manage the household expenses with her husband's income.

Sapna had developed a cough last year for which she went to a BMC / Municipal hospital near her house where after a sputum test and chest x-ray she was diagnosed with TB. She was given 6 or7 different types of tablets and had to take them on alternate days. (ek din chod kar). She took the treatment for 4 to 5 months and then stopped it as she felt better. But after stopping the medication she had mild cough which used to subside on its own. Her family has been keeping well and none of them have ever had any of his symptoms.

But since last one month she is suffering from cough which is more or less present during early morning and night, and she also has expectoration in which she has seen flecks of blood once or twice in a month.She also has low grade mild fever, on and off, which gets worse during the evening time. But since this problem started she feels a bit tired and also has lost some weight, as her clothes have gotten a bit loose. She does not suffer from any associated chest or body pain.She has tried some home remedies and cough syrup from a local chemist for the problem. However, the cough had not subsided.She has tried some home remedies and cough syrup from a local chemist for the problem. She has misplaced other medical records.

सपना (महिला)

सपना 38 साल की गृहणी है जिसने शहर में अपनी मौसी के पास रहकर 10वीं कक्षा तक पढ़ाई की है। सपना किराये के मकान में अपने पति और तीन बच्चों के साथ रहती है। सपना का पति 15000 रूपये महीना पर एक बड़े किराना दुकान में काम करता हैं। घर का गुजारा ठीक–ठाक चल रहा है। सपना के दो बच्चे सरकारी स्कूल में पढ़ते है जिनकी उम्र 13 (लड़का), 10 (लड़की) और तीसरा बच्चा 6 साल का है जो पास के प्ले स्कूल में जाता है।

सपना को पिछले साल खाँसी हो गयी थी जिसके लिये उसने घर के पास **बी एम सी / म्युनिसिपल अस्पताल** अस्पताल से इलाज कराया था। वहाँ पर थूक और एक्स–रे की जाँच कराने के बाद पता चला की उसे टी बी है।वहाँ से 6–7 तरह की दवाईयाँ मिलती थी जिनको एक दिन छोड़कर खाना होता था।उसने यह इलाज 4–5 महीनेलिया और फिर बन्द कर दिया क्योंकि उसे लगा कि उसे आरामआ गया है।इलाज छोड़ने के बाद सपना को बीच–बीच में खाँसी हो जाती थी मगर वह अपने आप ही ठीक हो जाती थी। उसकेपति और बच्चों का स्वास्थ्य अच्छा है और किसी को भी कोई लम्बी बिमारी नही रही।

लेकिनपिछले एक महीने से सपना को फिर से खाँसी और बुखार की परेशानी हो रही है।खाँसी के साथ बलगम भी आता है जिसमें पिछले महीने में एक–दो बार लाल रंग के धब्बे भी दिखाई दिये है।उसे हल्का बुखार है, जो चढ़ता–उतरता रहता है पर अक्सर शाम के समय ही ज्यादा रहता है। जब से उसे यह तकलीफ शुरु हुई है तब से उसे थकावट महसूस हो रही है। उसे लगता है कि उसका कुछ वजन कम हो गया है क्योंकि उसके कपड़े ढीले हो गये है। उसको किसी भी तरह का छाती और बदन दर्द नहीं है। सपना ने पिछले महीने कैमिस्ट से खाँसी का सिरपऔर कुछ घरेलू उपचार किये जिससे उसकी खाँसी में कुछ आराम नहीं आया। उसे लग रहा है कि अस्पताल वाले उसे इधर से उधर चक्कर लगवायेंगे,

इसलिए उसने प्राइवेट डॉक्टर से सलाह लेने के बारे में सोचा। उसके पास थूक की जाँच की रिपोर्ट है लेकिन बाकी मेडिकल रिकार्ड उससे कहीं खो गये है।सपना किसी भी प्रकार का नशा नहीं करती।

सपना (महिला)

सपना एक ३८ वर्षीय गृहिणी आहेत ज्यांनी शहरामध्ये आपल्या मावशीकड़े राहून १०वी इयत्ते पर्यंतचे शिक्षण पूर्ण केले. सपना भाड्याच्या घरात आपल्या पति अणि तीन मुलांसोबत राहतात. सपने चे पति १५००० रूपये प्रति महिनावर एक मोठ्या किराणा दुकानात काम करतात. घराचं गुजरण पण ठीक चालले आहे. सपनाची दोन मुले बी एम सी / म्युनिसिपल शाळेत शिकत आहेत ज्यांची वयं १३ (मुलगा), १० (मुलगी) आणि तीसरा मुलगा 6 वर्षांचा जवळच्या प्ले स्कूल मध्ये जात आहे.

सपना ह्यांना मागील वर्षी खोकला झाला होता ज्यासाठी त्यांनी त्यांच्या घराजवळच्या बी एम सी / म्युनिसिपल रुग्णालयात उपचार घेतले होते. तिकडे थुंकी आणि एक्स-रे च्या तपासणी केल्यानंतर कळलं कि त्यांना टी बी आहे. तिकडून ६-७ प्रकारच्या औषध-गोळ्या मिळत होत्या, ज्यांना एक दिवस सोडून घ्यायचं असायचं. त्यांनी हा उपचार ४-५ महिने घेतला आणि नंतर बंद केला कारण त्यांना बरं (आराम) वाटू लागले होते. उपचार बंद केल्यानंतर सपना ह्यांना अधून-मधून खोकला येत असे पण तो आपोआपच ठीक होत होता. सपना ह्यांच्या पतिचे आणि मुलांचे आरोग्य चांगले आहे आणि कोणालाही दीर्घकालीन आजार नाही आहे.

पण मागील एका महिन्यापासून सपना ह्यांना परत खोकला आणि तापाचा त्रास होऊ लागला आहे. खोकल्याबरोबर खाकरा (बेडका) पण येतोय ज्यामध्ये मागील महिन्यात एक-दोन वेळा लाल रंगाचे धब्बे पण दिसले होते.खोकल्याबरोबर त्यांना हलकासा ताप चढतो-उतरतो, पण अनेकदा संध्याकाळच्या वेळी ताप जास्ती होतो. जेव्हापासून हा त्रास सुरु झाला तेव्हापासून थकवा जाणवत आहे. त्यांना असा वाटतंय कि त्यांचा वजन थोडे कमी झालंय कारण त्यांना त्यांचे कपडे सैल झाले आहेत. त्यांना ह्या त्रासामध्ये कुठल्याहीप्रकारे छाती किंवा अंगदुखी नाही झाली आहे.सपनाने मागील महिन्यात केमिस्टकडून खोकल्यासाठी सिरप अणि काही घरगुती उपचार केले तरीसुद्धा त्यांच्या खोकल्याला काही आराम मिळाले नाही. त्यांना असं वाटतंय कि रुग्णालयात त्यांना इकडून तिकडे चक्कर मारायला लावतील, म्हणून त्यांनी प्रायवेट डॉक्टरांकडे सल्ला घेण्याचा विचार केला. त्यांच्याकडे थुंकीच्या तपासणीचे रिपोर्ट आहे पण त्यांचे बाकीचे मेडिकल रिकार्ड त्यांच्याकडूनच हरवले आहेत. सपना कोणत्याही प्रकारचा नशा करीत नाही.

Opening statement: "Doctor, I am suffering from a bad cough – One year ago I had got treatment in the BMC / Municipal hospital, and it had got better. But now again having cough again. I went back to the same hospital and they did sputum test." Can you see me.

[Note:- If asked, SP to present with the sputum smear report that is positive for acid fast bacillus]."

आपे निंग स्टेटमेटं :"डॉक्टर साहब, मुझे बहुत खा<सी हो रही है,. एक साल पहले **बी एम सी / म्युनिसिपल अस्पताल**में इलाज करवाया थाठीक हो गया था/गई थी। अब फिर स` खा<सी शुरू हो गई है। मै दाे ाारा अस्पताल में गया था/गई थी उन्होंने ो फिर थूकजा<च कराई है" आप मुझे देख लीजिये।
नोटः–पूछने पर ही, SP को एसिड फास्ट बेसिलस की पॉजिटिव स्पूटम सिमियर रिर्पोट दिखानी है)"

ओपनिंग स्टेमेंट : डॉक्टरसाहेब, मला खूप खोकला झाला आहे. एक वर्षापूर्वी मी बी एम सी / म्युनिसिपल रुग्णालयात उपचार घेतला होता आणि बरा झालो होतो \ बरी झाली होती. आता परत खोकला सुरु झाला आहे. मी पुन्हा त्याच रुग्णालयात गेलो \ गेली आणि त्यांनी माझी थुंकी ची तपासणी करून घेतली.

(नोट : विचारल्यावरच, एस पी नी एसिड फास्ट बेसिलस चा पोजिटीव स्मीयर रिपोर्ट दाखवावा)

<u>History questions asked by the provider and their answers</u>

<u>प्रोवाइडर द्वारा पूछे गये हिस्ट्री सवाल और उनके जवाब</u>

<u>प्रोवायडरद्वारा विचारले गेलेले हिस्ट्रीचे प्रश्न आणि त्यांचे उत्तर</u>

Q 1: What is the duration of cough?
प्रश्न 1: खाँसी कब से हो रही है?
प्रश्न 1: खोकला कधीपासून आहे?
Ans1: One Month.
उत्तर 1: एक महीने से।
उत्तर 1 : एका महिन्यापासून

Q 2 : When you had cough earlier you went to a BMC / Municipal hospital?
प्रश्न 2: जब पिछली बार आपको खाँसी हुई थी तब आप बी एम सी / म्युनिसिपल हॉस्पिटल गये थे?
प्रश्न 2. जेव्हा मागील वेळा आपल्याला खोकला झाला तेव्हा आपण बी एम सी / म्युनिसिपल रुग्णालयात गेले होतात का?
Ans 2: Yes, I did go there.
उत्तर 2: हाँ, मैं वहाँ गया था।
उत्तर 2: हो, मी गेलो होतो

Q 3: Did you get any testsfor the previous episode?
प्रश्न 3: पिछली बार वहाँ आपने कोई टेस्ट करवाये थे?
प्रश्न 3: मागच्या वेळा तिथे आपण कुठली टेस्ट केली होती का?
Ans 3: Yes, sputum test and a chest x-ray
उत्तर 3: हाँ, थूक की जाँच और छाती का एक्स–रे करवाया था।
उत्तर : हो, थुंकीची तपासणी आणि छातीचा एक्स-रे करून घेतला होता?

Q4: Did they tell you what you hadfor the previous episode?
प्रश्न 4: पिछली बार उन्होने बताया था कि आपको क्या हुआ है?
उत्तर 4: मागील वेळा आपल्याला त्यांनी सांगितलं होतं का आपल्याला काय झालं होते ते?
Ans 4: Yes, they told me I had TB
उत्तर 4: हाँ, उन्होने बताया था कि मुझे टी बी है।

उत्तर4. हो, त्यांनी मला सांगितलं होतं कि मला टी बी आहे म्हणून

Q. 5: What treatment did you get?
प्रश्न 5: आपको क्या इलाज मिला था?
प्रश्न 5. आपल्याला काय उपचार मिळाला होता?
Ans 5: I was given 6 or 7 types of tablets.
उत्तर 5: मुझे 6–7 तरह की गोलियाँ मिलती थी।
उत्तर5. मला 6-7 प्रकारच्या औषधं-गोळ्या दिल्या होत्या

Q6: Do you know what these were?
प्रश्न 6: क्या आपको पता है वह कौन सी गोलियाँ थी?
प्रश्न 6: आपल्याला माहिती आहे का कि आपल्याला कोणती औषधं-गोळ्या दिल्या होत्या?
Ans 6: No, I don't.
उत्तर 6: नही, मुझे नही पता।
उत्तर 6: नाही, मला नाही माहिती
Q7: What was the dosage?
प्रश्न 7: दवाई की खुराक क्या थी?
प्रश्न 7. औषधांचा खुराक (डोस) काय होता?
Ans 7: I had to take them every alternate day.
उत्तर 7: मैं हर दूसरे दिन दवाई खाता था।
उत्तर 7: मला एक दिवस सोडून (एक दिवस आड) औषधं-गोळ्या घ्यायचे होते

Q 8:For how many months did you take the medicine?
प्रश्न 8: आपने कितने महीने दवाई खाई?
प्रश्न8: आपण किती महिने औषधं घेतली ?
Ans 8: 4 to 5 months.
उत्तर 8: 4–5 महीने।
उत्तर8: 4-5 महिने

Q 9:Did they tell you to stop?
प्रश्न 9: क्या उन्होंने दवाई बन्द करने के लिये कहा था?
प्रश्न 9 : आपल्याला त्यांनी औषधं घ्यायाला बंद करायला सांगितली का?
Ans 9: No, but I was feeling much better - so I thought I did not need the medicines any more - they create garmi.
उत्तर 9: नहीं, मुझे अच्छा महसूस होने लगा था इसलिये मैंने सोचा कि और दवाईयों की जरुरत नहीं है, वो गर्मी करेंगी।
उत्तर 9 : नाही, पण मला बरं वाटायला लागलं म्हणून मी विचार केला कि जास्ती औषधांची जरुरत नाही - ते गर्मी वाढवतील

Q10: Do you have the sputum report or the x-rayfrom the previous episode?
प्रश्न 10: क्या आपके पास पिछली बार केथूक जाँच और एक्स–रे की रिपोर्ट है?
प्रश्न 10 : आपल्याकडे मागच्या वेळेचे थुंकीची तपासणी आणि एक्स-रे रिपोर्ट आहे का?
Ans 10: No, I misplaced all the documents from the hospital.
उत्तर 10: नहीं, अस्पताल के सारे कागज मुझसे कहीं खो गये है।

उत्तर 10: नाही, रुग्णालयाचे सगळे कागदपत्रे हरवली आहेत

Q 11: So your cough got better and now it is back?
प्रश्न 11: क्या तब आपकी खाँसी ठीक हो गई थी औरअब दोबारा हो गई है?
प्रश्न 11 : आपला खोकला ठीक झाला होता आणि आता परत झाला का?
Ans 11: Yes.
उत्तर 11: हाँ।
उत्तर 11. हो

Q 12: Are you producing sputum (bulgam)?
प्रश्न 2: क्या आपको बलगम बनती है?
प्रश्न 2 : खाकरा/बेडका तयार होतो का?
Ans 2: Yes
उत्तर 2: हाँ।

Q 13: Does the sputum contain blood?
प्रश्न 13: क्या आपके बलगम में खून आता है?
प्रश्न 13 : खाकरा/बेडका/थुंकी मध्ये रक्त पडते का?
Ans13: Yes, saw small flecks of blood once or twice in the last month.
उत्तर13: हाँ, पिछले महीने में एक–दो बार खून के हल्के–हल्के धब्बे आये थे।
उत्तर 13: हो, मागील महिन्यात एक-दोन वेळा रक्ताचे धब्बे दिसले होते

Q 14: Do you get fever?
प्रश्न 14: क्या आपको बुखार है?
प्रश्न 14 : आपल्याला ताप आहे का?
Ans 14: Yes.
उत्तर 14: हाँ।
उत्तर 14. हो

Q 15: How long have you had fever?
प्रश्न15: आपको बुखार कब से है?
प्रश्न 15. आपल्याला ताप कधीपासून आहे?
Ans 15: One Month.
उत्तर15: एक महीने से।
उत्तर 15: एका महिन्यापासून

Q 16: What type of fever do you have?

प्रश्न5: बुखार कैसा रहता है?

प्रश्न 16 : ताप कसा असतो?

Ans 16: Low grade (mild), on and off, more during evening times.

उत्तर 16: हल्का बुखार चढ़ता उतरता रहता है, लेकिन ज्यादातर शाम को होता है।

उत्तर 16. हलकासा ताप चढतो-उतरतो, पण जास्तीकरून संध्याकाळी वाढतो

Q 17: Do you get any chest pain?

प्रश्न17: क्या आपकी छाती में दर्द होता है?

प्रश्न 17: आपल्या छाती मध्ये दुखतंय का?

Ans 17: No

उत्तर 17: नहीं।

उत्तर 17: नाही

Q18: Any loss of appetite?

प्रश्न18: भूख में कोई कमी?

प्रश्न 18: भूक कमी झाली आहे का?

Ans 18: Yes, loss of appetite.

उत्तर 18: हाँ, भूख तो कम लगती है।

उत्तर 18. हो, भूक कमी लागते.

Q19: Have you lost weight?

प्रश्न19: क्या वजन कम हुआ है?

प्रश्न 19: आपलं वजन कमी झाले आहे का?

Ans 19: I think so; my clothes have become a bit loose.

उत्तर 19: हाँ, मुझे लगता है क्योंकि मेरे कपड़े ढीले हो गये हैं।

उत्तर 19 : हो, मला वाटतंय कारण माझे कपडे मला सैल होत आहेत

Q20: Do you smoke?

प्रश्न20: क्या आप बीड़ी/सिगरेट पीते हैं?

प्रश्न20: विडी-सिगारेट पिता का?

Ans 20: Yes, I smoke beedis. [in case of male SP]

उत्तर 20: हाँ, मैं बीड़ी पीता हूँ। (मेलSP के लिये)

No [in case of females SP] नहीं (फीमेल SP के लिये)

Daniels B, *et al. BMJ Global Health* 2022; 7:e009657. doi: 10.1136/bmjgh-2022-009657

उत्तर 20: हो, मी बीडी/सिगारेट (पुरुष एस पी साठी) पितो, नाही (स्त्री एस पी साठी)

Q 21: How manybeedis in a day?
प्रश्न 21: एक दिन में कितनी पीते हो?
प्रश्न 21: एका दिवसात किती बीडी/सिगारेट पिता?
Ans 21: 4-5beedis, I guess
उत्तर 21: अंदाजन, 4 से 5 बीड़ी।
उत्तर 21 : अंदाजे, ४ ते ५ बीडी/सिगारेट

Q 22: Since when have you been smoking beedis?
प्रश्न 22: कब से बीड़ी पी रहे हो?
प्रश्न 22 : आपण कधीपासून बीडी/सिगारेट पीत आहात?
Ans 22: Since the last 10 or 12 years.
उत्तर 22: पिछले 10 या 12 सालों से।
उत्तर 22: मागील 10 ते 12 वर्षांपासून

Q 23: Do you drink alcohol?
प्रश्न 23: क्या आप शराब पीते ेम्æ
प्रश्न 23: आपण दारू पिता का?
Ans 23: Yes [in case of male] No [in case of female SP]
उत्तर 23: हाँ (मेल SP के लिये), नहीं (फीमेल SP के लिये)
उत्तर 23: हो, मी दारू (पुरुष एस पी साठी) पितो, नाही (स्त्री एस पी साठी)

Q 24: Does anyone in your family have TB?
प्रश्न24: क्या आपके परिवार में किसी को टीबी है?
प्रश्न 24 : आपल्या कुटुंबामध्ये कोणाला टी बी आहे का?
Ans 24: No.
उत्तर 24: नहीं।
उत्तर 24 : नाही

Q 25: Have you taken any medicines for your illness in last one month?
प्रश्न 25: क्या पिछले एक महीने में आपने इस तकलीफ के लिये कोई दवाई ली है?
प्रश्न 25 : मागच्या एका महिन्यात आपल्याला झालेल्या ह्या त्रासाबद्दल आपण कोणती औषधं घेतली का?
Ans 25: Took home remedies and took cough syrup from a chemist.
उत्तर25: कुछ घरेलू उपचार किये और कैमिस्ट से कफ सिरप खरीदा था।
उत्तर 25 : काही घरगुती उपचार घेतले होते आणि केमिस्ट कडून कफ सिरप विकत घेतलं होतं

Q26: Do you have diabetes?

प्रश्न 26: क्या आपको शुगर ३æ

प्रश्न 26: आपल्याला साखरेचा त्रास आहे का?

Ans 26: I do not know

उत्तर 26: जी,पता नहीं।

उत्तर 26: माहित नाही

Q 27: Do you have hypertension?

प्रश्न 27 : क्या आपको हाईपरटेंशन है?

प्रश्न 27 : आपल्याला हायपरटेंशन/रक्तदाबाचा त्रास आहे का?

Ans 27: I do not know

उत्तर 27: जी,पता नहीं।

उत्तर 27: माहित नाही

Q28 Do you have HIV-AIDS?

प्रश्न28: क्या आपको HIV-AIDS है?

प्रश्न28: आपल्याला HIV-AIDS आहे का?

Ans 28:I do not know

उत्तर 28: जी,पता नहीं।

उत्तर 28: माहित नाही

Q29: Have you ever been tested for HIV-AIDS, Diabetes and High BP/Hypertension?

प्रश्न29: क्या आपने कभी एच आई वी–एड्स, शुगर और हाई बी पी/हाईपरटेंशन बिमारियों की जाँच या टेस्ट करवाया है?

प्रश्न 29 आपण कधी एच आय वी-एड्स, शुगर आणि हाय बी पी/हायपरटेंशन च्या तपासण्या केल्या होत्या का?

Ans 29: Not been tested.

उत्तर 29: कभी टेस्ट नहीं करवाया।

उत्तर 29 : कधी टेस्ट केल्या नाही

Important instructions to be remembered by SP

महत्वपूर्ण बातें जो एस पी को याद रखनी है

एस पी साठी महत्वाच्या सूचना

1.	ISERDD staff will identify the facility and/or the provider for the SP, where the SP will present his case alone.<br>ISERDDस्टाफ फील्ड मेंSPको प्रोवाइडर का क्लीनिक बतायेगा जहाँSPको अकेले अपना केस करना है।<br>ISERDD स्टाफ फील्ड मध्ये SP ला प्रोवायडरचे क्लिनिक सांगतील जिकडे SP ला एकटच आपली केस सादर करायची आहे।
2.	SP should remember if the facility and/or the provider was the correct one. (for example, it was the correct location, but the name board did not match the name given or if the assigned provider was not there and the SP saw another provider)<br>SPको याद रखना है कि वो सही प्रोवाइडर के क्लीनिक गये थे? (उदाहरण के लिए, जगह सही थी लेकिन बोर्ड पर लिखा नाम दिये गये नाम से मैच नहीं कर रहा था।<br>SPनी लक्षात ठेवायचे आहे कि ते योग्य ते प्रोवायडरच्या क्लिनीक मध्ये गेले होते का? (उदाहरणासाठी जागा बरोबर होती पण बोर्डावरचे नाव दिलेल्या नावाशी मैच होत नव्हतं)
3.	SP must remember if the provider carried out any of the following examination?<br>SP को याद रखना है कि प्रोवाइडर ने निम्नलिखित में से कोई परीक्षण किये?<br>SPनी लक्षात ठेवायचे आहे कि प्रोवायडरनी खालीलपैकी कोणत्या तपासण्या केल्या?<br>• Pulse rate नब्ज़ की दर नाडीचे स्पंदन मोजणे<br>• Respiratory rate साँस की दर श्वसन दर मोजणे<br>• Auscultation of Chest(Did the provider comment on the auscultation) ह्रदय, फेफड़ों की गति को सुनना (क्या प्रोवाइडर ने ऑस्कलटेशन करते समय कुछ कहा जैसे आवाज साफ है या अन्य) ह्रदय फुफ्फुसे अंतर्गत होणाऱ्या आवाज ऐकून तपासणे (प्रोवायडर ने ऑस्कलटेशन करताना काय सांगितलं का जसे आवाज साफ आहे किंवा अन्य)<br>• Blood Pressure ब्लड प्रेशर ब्लड प्रेशर / रक्तदाब मोजणे<br>• Temperature बुखार मापना – थर्मामीटर ताप मापन - थर्मामीटरने<br>• Throat examination गले का परीक्षण गळ्याचे परीक्षण<br>• Weight वजन मापना वजन मापनAbdomen palpitation पेट दबाकर परीक्षण पोटाचे दाबून परीक्षण करणे

4.	Did the provider recommend any investigations? क्या प्रोवाइडर ने निम्नलिखित जाँच कराने को कहा?<br>प्रोवायडरनी खालीलपैकी कोणत्या तपासण्या/चाचण्या करायला सांगितल्या?<br>(If yes, SP should ask provider to write the name of the test and the laboratory. And hand over the document to an ISERDD staff.) *Write the specific name of lab given by the provider and if no put (-99).*<br>(यदि हाँ, तो SPको प्रोवाइडर से टेस्ट और लैब का नाम लिखित में लेना है और उस पर्चे को ISERDD स्टॉफ को सौंप देना है) *अगर प्रोवाइडर ने किसी विशेष लैब का नाम दिया है तो वह लिखें, यदि नहीं तो (-99) भरें।*<br>(जर हो तर, SP नी प्रोवायडरकडून टेस्ट आणि लैबचे नाव लिहून घ्यायचे आहे आणि त्या कागदाला ISERDD स्टाफला द्यायचे आहे) जर प्रोवायडरनी विशेष लैब चे नाव सुचवले तर ते लिहून घ्या, जर नाही सांगितले तर (-99) भरा<br>• Chest X-Ray छाती का एक्स-रे छातीचा एक्स-रे<br>• CT Scan सी टी स्केन सी टी स्कॅन<br>• Blood- Total count, differential count- ESR रक्त-टोटल काउंट, डिफरेंशियल काउंट-ई एस आर रक्त टोटल काउंट डिफरेंशियल काउंट - ई एस आर<br>• Blood- HIV test रक्त-एच आईवी टेस्ट रक्त एच आय वी टेस्ट -<br>• Blood- Diabetes test रक्त-शुगर टेस्ट रक्त शुगर टेस्ट -<br>• Blood- TB Gold रक्त – टी बी गोल्ड रक्त टी बी गोल्ड टेस्ट -<br>• Blood-TB ELISA रक्त – टी बी ऐलाइजा रक्त टी बी ऐलाइजा -<br>• Sputum smear examination (Sputum AFB) स्पूटम स्मियर एक्जामिनेशन स्पूटम स्मियर एक्जामिनेशन स्पूटम ए एफ बी /<br>• Sputum GeneXpert test स्पूटम जीनएक्सपर्ट टेस्ट स्पूटम जीनएक्सपर्ट टेस्ट<br>• Sputum culture स्पूटम कल्चर स्पूटम कल्चर<br>• Mantoux Test मॉनटुक्स टेस्ट मौनटूक्स टेस्ट<br>• TB- PCR (not specific) टी बी - पी सी आर (विशिष्ट नहीं है) टी बी - पी सी आर (विशेष नाही आहे)<br>• Drug susceptibility test (culture, gene Xpert, line probe assay) ड्रग स्सेप्टिबिलिटी टेस्ट (कल्चर, जीनएक्सपर्ट, लाइन प्रोब एस्से) ड्रग स्सेप्टिबिलिटी टेस्ट (कल्चर, जीनएक्सपर्ट, लाईन प्रोब एस्से)
5.	Did the provider give you any vouchers or referral coupons?<br>क्या प्रोवाइडर ने SP को कोई वाऊचर या रेफरल कूपन दिया? प्रोवायडरनी SPला कुठले वाऊचर/रेफेरल कूपन दिले का?<br>(If yes, SP should retain the voucher or referral coupons and hand over to any ISERDD staff. On the form, supervisors will write the voucher number and name from the stamp.)<br>(यदि हाँ, तो SP को वाऊचरया रेफरल कूपन सभांल कर रखना है और ISERDDस्टाफ के हाथों में सौंपना है)<br>(जर हो, तर SP नी वाऊचर/रेफेरल कूपन सांभाळून ठेवायचे आहे आणि ISERDD स्टाफ च्या हातात द्यायचे आहे)<br>• Treatment उपचार उपचार<br>• Chest X-Rayछाती का एक्सरे छातीचा एक्स-रे<br>• GeneXpertजीनएक्सपर्ट टेस्ट जीनएक्सपर्ट टेस्ट<br>Please write voucher or referral coupons number and name on the stamp.<br>कृप्या वाऊचर या रेफरल कूपन का नम्बर तथा स्टैम्प पर जो नाम लिखा है उसे लिखें।<br>कृपया वाऊचर किंवा रेफरल कूपन चा नंबर आणि स्टैम्पवर जे नाव लिहिलेलं असेल ते लिहा

6.	SP must remember if personal details (Name, Mobile, Address) were given to the provider. If so, what was the full name the SP gave? (Some providers who work with the PPIA will collect this information if they identify the SP as a presumptive TB case, refer you to a lab for sputum or CXR, or refer you to certain providers.)<br>SPको याद रखना है कि उसनेप्रोवाइडर को क्या निजी जानकारी दी। जैसे नाम, मोबाइल नम्बर, पता। यदि दी, तो एस पी ने अपना पूरा नाम क्या बताया? (PPIA के साथ काम करने वाले कुछ प्रोवाइडरइस जानकारी को इकट्ठा करेंगे यदि वह पहचान ले कि आपएक संभावितTBकेस हो तो वह आपको छाती एक्स–रे या थूक जाँच के लिए लैब में रेफर या चुने हुए प्रोवाइडर के पास रेफर करेंगें)<br><br>SPनी लक्षात ठेवायचे आहे कि त्यांनी कोणती वैयक्तिक माहिती प्रोवायडरला दिली आहे. जसा आपला नाव, मोबाईल नंबर, पत्ता, जर दिली असेल, तर SPनी आपलं पूर्ण नाव काय सांगितलं? (PPIA च्या बरोबर काम करणारे काही प्रोवायडरर्स आपली माहिती एकत्रित करतील आणि जर त्यांना वाटलं कि आपण एक संभावित TB केस आहात तर ते आपल्याला छातीचा एक्स-रे किंवा थुंकीची तपासणीसाठी लैब मध्ये रेफर किंवा निवडलेले प्रोवायडरकडे जाण्यासाठी रेफर करतील)
7.	SP must remember if someone at the facility completed a registration form to register the SP's personal details such as with a call center. (Some providers who work with the PPIA will make a call to register patients.)<br>SP को याद रखना है कि प्रोवाइडर ने SP की व्यक्तिगत जानकारी रजिस्टर करने के लिए फोन एक कॉल सेंन्टर को फोन किया।<br>(PPIA के साथ काम करने वाले कुछ प्रोवाइडर पेशेंट को रजिस्टर करने के लिए फोन करेंगे।)<br><br>(SPनी लक्षात ठेवायचे आहे कि जर कोणी फैसिलीटीमध्ये कोणी SPची वयतिक्त माहितीची नोंदणी करण्यासाठी कॉलसेंटरला कळविले (काही प्रोवायडर जे PPIA बरोबर काम करतात ते पेशंट रजिस्टर करण्यासाठी कॉल करतील)
8.	ISERDD staff to mark which of the following tests was recommended by a networked provider.(Refer to networked provider/lab list)<br>जो भी टेस्ट नेटवर्क प्रोवाइडर द्वारा करवाने के लिए बोला गया है उसेISERDD स्टॉफ को फार्म में मार्क करना है।(नेटवर्क प्रोवाइडर/लैब पर रेफर किया)<br>जी कोणतीही टेस्ट नेटवर्क प्रोवायडरद्वारा करण्यातही सांगितली आहे ती टेस्ट ISERDD स्टाफने फॉर्ममध्ये मार्क करायच्या आहेत (नेटवर्क प्रोवायडर / लैब ला रेफर केलं)
9.	SP must remember if the provider gave any diagnosis.<br>SPको प्रोवाइडर द्वारा दिये गये डाइग्नोसिस को याद रखना है।<br>SPनी प्रोवायडरने दिलेल्या डायग्नोसिस ला लक्षात ठेवायचे आहे?
10.	SP must remember if the provider recorded the information he took from you.<br>SPको यह ध्यान रखना है कि प्रोवाइडर ने आपसे जो जानकारी ली उसको कहीं लिखकर रखा।<br>SPने लक्षात ठेवायचे आहे कि प्रोवायडर ने आपण दिलेली जी माहिती घेतली ती कुठे लिहून नोंदली?:
11.	SP should remember if the provider referred to: SPको याद रखना है यदि प्रोवाइडर रेफर करता है तो :<br>जर प्रोवायडरने रेफर केलं तर SPनी खालील गोष्टी लक्षात ठेवायचे आहेत<br>• a private provider/ private hospitalप्राईवेट प्रोवाइडर/प्राईवेट अस्पताल प्रायवेट प्रोवायडर / प्रायवेट रुग्णालय<br>• a BMC / Municipal hospitalसरकारी अस्पताल बी एम सी / म्युनिसिपल रुग्णालय<br>• DOTS centreडॉट्स सेन्टर डॉट्स सेंटर<br>• Other locationअन्य स्थान अन्य जागा

	and include the name of the provider or health facility you were referred to:<br>जहाँ रैफर किया है उस प्रोवाइडर या हेल्थ फैसिलिटी का नाम शामिल करे :<br>आणि जिकडे रेफर केलं त्या प्रोवायडर किंवा फैसिलीटीचे नाव सामील करा
12.	SP should refuse any injections/ invasive tests performed by the provider during this encounter but note down details of what was offered/suggested.<br>SPको इस बातचीत के दौरान किसी भी तरह का इन्जेक्शन/इन्वेसिव टेस्ट लेने से इन्कार करना है लेकिन ऐसे किसी भी सुझाव को नोट करके बताना है।<br>SP नी संभाषण दरम्यान कुठल्याही प्रकारचे इंजेक्शन / इन्वेसिव टेस्ट घ्यायला नकार द्यायचा आहे आणि अश्या प्रकारच्या सूचना नोट करून ठेवायच्या आहेत
13.	If the provider asks any question that is not in the list then the SP should say no or deflect by either saying I don't know or I don't remember as is appropriate to the context.<br>यदि प्रोवाइडर कोई ऐसा सवाल पूछता है जो लिस्ट में शामिल नहीं है तो SP को कहना है मुझे पता नहीं या याद नहीं आ रहा (जो उस समय उचित लगे)<br>जर प्रोवायडर आपल्याला असा प्रश्न विचारतात जो लिस्ट मध्ये सामील नाही आहे तर SPनी सांगायचे आहे कि मला माहित नाही किंवा आठवत नाही (जे त्या वेळी उचित वाटेल ते)
14.	SP must collect prescription and/or any medicines given by the provider.<br>SPको प्रोवाइडर द्वारा दी गयी दवाई या दवाई का पर्चा अवश्य लेना है।<br>SPनी प्रोवायडरने दिलेल्या औषधं आणि औषधांच कागद घ्यायचा आवश्यकचं आहे
15.	SP should get the prices of the prescribed medicines, labs or consultation fees from the provider.<br>SP को प्रोवाइडर द्वारा पर्चे पर लिखी दवाई, लैब या कन्सल्टेशन फीस की जानकरी को याद रखना है।<br>SPनी प्रोवायडरद्वारा औषधांसाठी दिलेला कागदावर लिहलेली औषधं, लैब किंवा कन्सलटेशन फीज ची माहितीला लक्षात ठेवायचे आहे
16.	Supervisor /SP should get the prices, brand and generic names of the prescribed medicines.<br>Supervisor/SP को प्रोवाइडर द्वारा लिखी दवाई का मूल्य, ब्रांड तथा जेनरिक नाम का पता करना है।<br>Supervisor/ SP ला प्रोवायडरद्वारा दिले गेलेल्या औषधांचे मूल्य, ब्रांड आणि जेनरिक नावांची माहिती गोळा करायची आहे<br>• Fill all unlabelled FIRST खुली दवाईयाँ पहले लिखें खुली औषधं पहिले लिहा<br>• In brand: write Unlabelled ब्रॉण्ड में अनलेबल्ड लिखें ब्रांड मध्ये अनलेबल्ड लिहा<br>• In generic: describe the color + shape जेनेरिक में दवाई का आकार व रंग लिखें जेनेरीक मध्ये रंग आणि आकार लिहा
17.	Prescriptions and pills given must be preserved for analysis.<br>SPको पर्चा और दवाईयाँ एनालिसिस के लिये संभाल कर रखना है।<br>SPनी डॉक्टरांनी औषधांसाठी दिलेला कागद आणि औषधं-गोळ्या एनालीसिससाठी सांभाळून ठेवायच्या आहेत

Table A1. History question completion

Exams	*Public Dispensary*	*Public Hospital*	*Private Sector*
Pulse	13.6%	13.2%	59.1%
Auscultation	28.7%	42.7%	92.7%
Provider commented on auscultation	50.0%	66.7%	66.2%
Temperature	0.0%	1.3%	20.2%
Throat Exam	13.6%	2.6%	40.9%
Blood Pressure	2.3%	5.3%	65.5%
Weight	4.5%	0.0%	37.9%
Abdomen palpitation	1.1%	0.0%	33.5%
Case 1 Questions	*Public Dispensary*	*Public Hospital*	*Private Sector*
Duration of Cough	95.5%	92.1%	99.0%
Sputum	54.5%	57.9%	86.5%
Past TB	29.5%	60.5%	43.4%
Family TB	15.9%	34.2%	24.7%
Blood in Sputum	15.9%	10.5%	27.9%
Cough Throughout Day	20.5%	15.8%	61.7%
Fever	79.5%	78.9%	94.6%
Fever Type	29.5%	34.2%	73.7%
Family or Family with Similar Symptoms	2.3%	10.5%	13.2%
Chest Pain	11.4%	18.4%	37.4%
Loss of Appetite	13.6%	23.7%	55.0%
Lost Weight	13.6%	21.1%	51.7%
Wheezing	0.0%	0.0%	2.5%
Difficulty Breathing	4.5%	26.3%	30.8%
Smoking	6.8%	2.6%	25.7%
Alcohol History	9.1%	2.6%	16.3%
Taken Medicines for Illness	38.6%	26.3%	71.2%
Diabetes	2.3%	15.8%	20.9%
HIV/AIDS	0.0%	2.6%	2.3%
Age	86.4%	76.3%	83.2%
Duration of Cough	93.2%	94.7%	96.8%
Sputum	38.6%	65.8%	86.8%
Case 2 Questions	*Public Dispensary*	*Public Hospital*	*Private Sector*
Medication Taken Last Month for Present Illness	18.2%	15.8%	33.2%
Visited Governmental Hospital for Previous Illness	52.3%	42.1%	32.8%
Treatment for Previous Illness	50.0%	57.9%	31.6%
Sputum or X-Rays Done for Previous Illness	15.9%	23.7%	33.6%
Diagnosis Given by Governmental Hospital	40.9%	55.3%	43.2%
Past TB Treatment	61.4%	57.9%	50.4%
For How Long Past TB Treatment	81.8%	86.8%	52.8%
Reason for Stopping	75.0%	71.1%	45.2%
Previous Treatment Medical Records	31.8%	36.8%	34.8%
Blood in Sputum	2.3%	13.2%	16.9%
Cough Throughout Day	4.5%	21.1%	46.6%
Fever	43.2%	76.3%	87.6%
Fever Type	25.0%	39.5%	55.0%
Similar Symptoms Before	6.8%	2.6%	6.8%
Family TB	2.3%	10.5%	6.4%
Chest Pain	6.8%	13.2%	21.7%
Loss of Appetite	15.9%	18.4%	55.4%
Lost Weight	11.4%	18.4%	44.2%
Wheezing	0.0%	0.0%	5.6%
Difficulty in Breathing	2.3%	5.3%	28.5%
Smoking	11.4%	13.2%	33.3%
Alcohol	9.1%	7.9%	18.1%
Diabetes	4.5%	5.3%	29.7%
HIV/AIDS	2.3%	0.0%	0.0%
High Blood Pressure or Hypertension	2.3%	2.6%	22.9%
Presence of Children	4.5%	5.3%	14.1%
Age	81.8%	73.7%	79.9%

Daniels B, *et al. BMJ Global Health* 2022; 7:e009657. doi: 10.1136/bmjgh-2022-009657

Table A2. Regression comparison of public vs private sector

Comparison vs Private Sector	Difference	Standard Error	T-Statistic	P-Value	Lower 95% CI	Upper 95% CI
**Family 1**						
STCI Management: Public Dispensaries	-0.188	0.066	-2.864	0.004	-0.317	-0.059
STCI Management: Public Hospitals	-0.018	0.064	-0.283	0.777	-0.145	0.108
MCGM Protocol: Public Dispensaries	0.371	0.060	6.180	0.000	0.253	0.489
MCGM Protocol: Public Hospitals	0.336	0.071	4.768	0.000	0.198	0.475
Share of Questions: Public Dispensaries	-0.183	0.023	-8.002	0.000	-0.228	-0.138
Share of Questions: Public Hospitals	-0.152	0.028	-5.507	0.000	-0.206	-0.098
Chest X-Ray: Public Dispensaries	-0.257	0.065	-3.953	0.000	-0.385	-0.129
Chest X-Ray: Public Hospitals	-0.158	0.066	-2.388	0.017	-0.288	-0.028
Sputum AFB: Public Dispensaries	0.437	0.066	6.630	0.000	0.307	0.566
Sputum AFB: Public Hospitals	0.361	0.074	4.852	0.000	0.214	0.507
Xpert MTB/RIF: Public Dispensaries	-0.119	0.048	-2.470	0.014	-0.214	-0.024
Xpert MTB/RIF: Public Hospitals	-0.017	0.077	-0.218	0.827	-0.168	0.134
TB Suspicion: Public Dispensaries	0.152	0.065	2.327	0.020	0.024	0.281
TB Suspicion: Public Hospitals	0.209	0.054	3.860	0.000	0.102	0.315
Referred Away: Public Dispensaries	0.001	0.034	0.015	0.988	-0.065	0.067
Referred Away: Public Hospitals	0.030	0.031	0.978	0.329	-0.031	0.092
**Family 2**						
Started TB Treatment: Public Dispensaries	0.006	0.018	0.335	0.738	-0.029	0.041
Started TB Treatment: Public Hospitals	-0.016	0.009	-1.739	0.083	-0.035	0.002
Fluoroquinolones: Public Dispensaries	0.005	0.031	0.145	0.885	-0.057	0.066
Fluoroquinolones: Public Hospitals	-0.028	0.021	-1.321	0.187	-0.069	0.014
Other Antibiotic: Public Dispensaries	0.155	0.064	2.422	0.016	0.029	0.281
Other Antibiotic: Public Hospitals	0.242	0.069	3.487	0.001	0.105	0.378
Steroids: Public Dispensaries	-0.023	0.008	-2.788	0.006	-0.039	-0.007
Steroids: Public Hospitals	-0.022	0.008	-2.837	0.005	-0.037	-0.007
**Family 3**						
SP Subjective Rating (1-10): Public Dispensaries	0.186	0.203	0.914	0.361	-0.214	0.586
SP Subjective Rating (1-10): Public Hospitals	0.128	0.227	0.564	0.573	-0.319	0.575
Provider Used Cell Phone: Public Dispensaries	0.022	0.047	0.473	0.637	-0.071	0.116
Provider Used Cell Phone: Public Hospitals	-0.051	0.027	-1.868	0.063	-0.104	0.003
Other People Were In Room: Public Dispensaries	0.014	0.034	0.417	0.677	-0.053	0.081
Other People Were In Room: Public Hospitals	0.370	0.079	4.702	0.000	0.215	0.525
Provider Had A TV On: Public Dispensaries	-0.002	0.002	-0.977	0.329	-0.007	0.002
Provider Had A TV On: Public Hospitals	-0.003	0.003	-0.990	0.323	-0.008	0.003
SP Liked The Provider: Public Dispensaries	0.041	0.023	1.762	0.079	-0.005	0.088
SP Liked The Provider: Public Hospitals	0.011	0.027	0.409	0.683	-0.043	0.065
SP Would Go To This Provider: Public Dispensaries	0.052	0.024	2.193	0.029	0.005	0.099
SP Would Go To This Provider: Public Hospitals	0.022	0.027	0.810	0.418	-0.032	0.076
Provider Created A Private Environment: Public Dispensaries	0.049	0.045	1.090	0.276	-0.039	0.137
Provider Created A Private Environment: Public Hospitals	-0.493	0.063	-7.827	0.000	-0.617	-0.369
Provider Seemed Knowledgeable About Illness: Public Dispensa	0.043	0.057	0.759	0.448	-0.069	0.156
Provider Seemed Knowledgeable About Illness: Public Hospitals	-0.017	0.056	-0.301	0.763	-0.126	0.092
Provider Addressed Worries Seriously: Public Dispensaries	-0.033	0.062	-0.531	0.596	-0.155	0.089
Provider Addressed Worries Seriously: Public Hospitals	-0.012	0.060	-0.201	0.841	-0.130	0.106
Provider Explained SP Condition: Public Dispensaries	-0.085	0.019	-4.408	0.000	-0.123	-0.047
Provider Explained SP Condition: Public Hospitals	-0.016	0.038	-0.426	0.670	-0.091	0.058
Provider Explained SP Treatment Plan: Public Dispensaries	0.042	0.062	0.678	0.498	-0.080	0.164
Provider Explained SP Treatment Plan: Public Hospitals	-0.122	0.051	-2.410	0.016	-0.222	-0.023
**Family 4**						
Time Waiting (min): Public Dispensaries	-17.709	5.457	-3.245	0.001	-28.442	-6.977
Time Waiting (min): Public Hospitals	68.796	17.920	3.839	0.000	33.555	104.037
Amount Paid (INR): Public Dispensaries	-505.314	25.129	-20.109	0.000	-554.731	-455.897
Amount Paid (INR): Public Hospitals	-504.523	24.904	-20.259	0.000	-553.497	-455.549
Time with Provider (min): Public Dispensaries	-2.671	0.271	-9.842	0.000	-3.204	-2.137
Time with Provider (min): Public Hospitals	-2.096	0.450	-4.655	0.000	-2.982	-1.211

Notes: This figure reports the results of a series of regressions using both the public hospital interactions (N=76) and private hospital interactions (N=633). Each point reports the real-valued coefficient for the indicator variable of the private sector and the corresponding confidence interval. Estimates are controlled for individual SP ID and the case scenario; standard errors are clustered by facility. The measures are grouped into four families for illustrative purposes.

Figure A1. Case management with private sector split by MD specialist qualifications

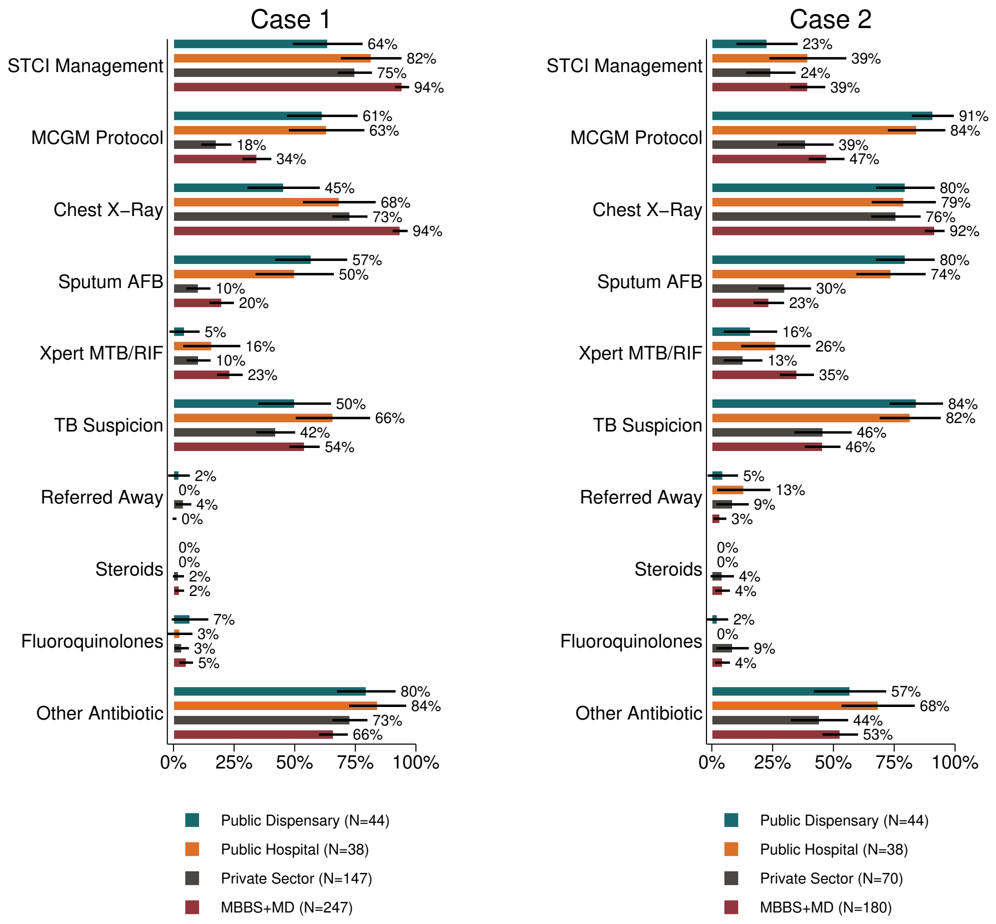

Notes: This figure reports the proportion of interactions in each study strata that resulted in the indicated outcome for an SP presenting Case 1 or Case 2 as indicated. The STCI management measure is defined as each Case 1 interaction that received a referral, a chest X-ray, a sputum AFB test, or an Xpert CBNAAT test or other drug-susceptibility test; and for each Case 2 interaction that received a referral or an Xpert CBNAAT test or other drug-susceptibility test. TB: tuberculosis, AFB: acid-fast bacillus; Xpert MTB/RIF: CBNAAT mycobacterium tuberculosis / rifampicin sensitivity testing. Medications from each interaction were ex-post coded by name to correspond to ATC code classifications. Fluoroquinolone antibiotics were defined as ATC codes beginning with J01M; steroids were defined as ATC codes beginning with H02, R01, or R03.

Daniels B, *et al. BMJ Global Health* 2022; 7:e009657. doi: 10.1136/bmjgh-2022-009657

Figure A2. Regression comparison of public vs private sector non-MD providers

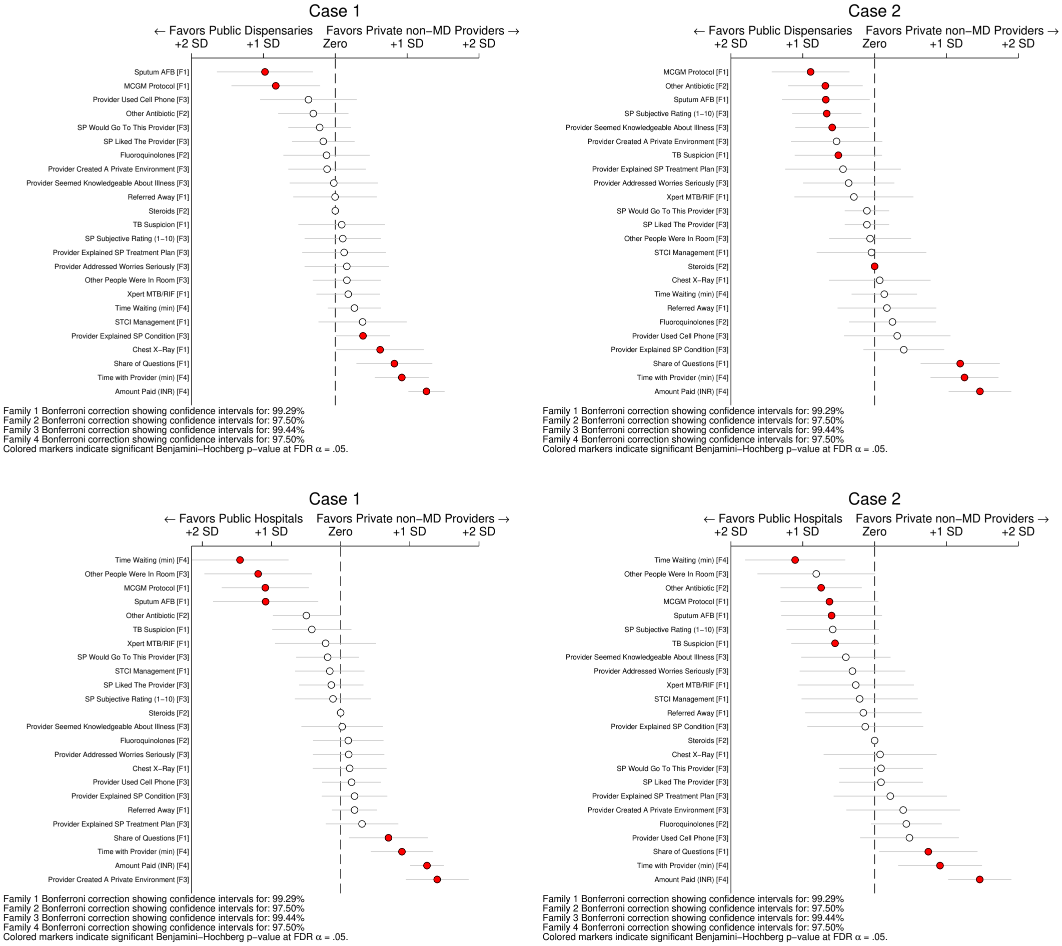

Notes: This figure reports the results of a series of regressions using both separately comparing the different types of public sector facilities with private sector non-MD providers. Each point reports the coefficient for the indicator variable of the private sector and the corresponding confidence interval. All measures are standardized to mean 0 and standard deviation 1 for illustration purposes. Estimates are controlled for individual SP ID and the case scenario; standard errors are clustered by facility. The measures are grouped into four families: Family 1 are the appropriate clinical management outcomes; Family 2 are the medication use outcomes; Family 3 are the subjective experience measures; and Family 4 are the unassociated characteristics. Each family's confidence intervals are extended to the appropriate Bonferroni interval for the number of simultaneous hypothesis; and the p-values are considered significant if they satisfy the Benjamini-Hochberg step-up procedure (including values that may not be considered significant under their own Bonferroni interval). Measures are sorted by the magnitude and direction of the estimated coefficient.

Daniels B, *et al. BMJ Global Health* 2022; 7:e009657. doi: 10.1136/bmjgh-2022-009657

Figure A3. Regression comparison of public vs private sector MD specialists

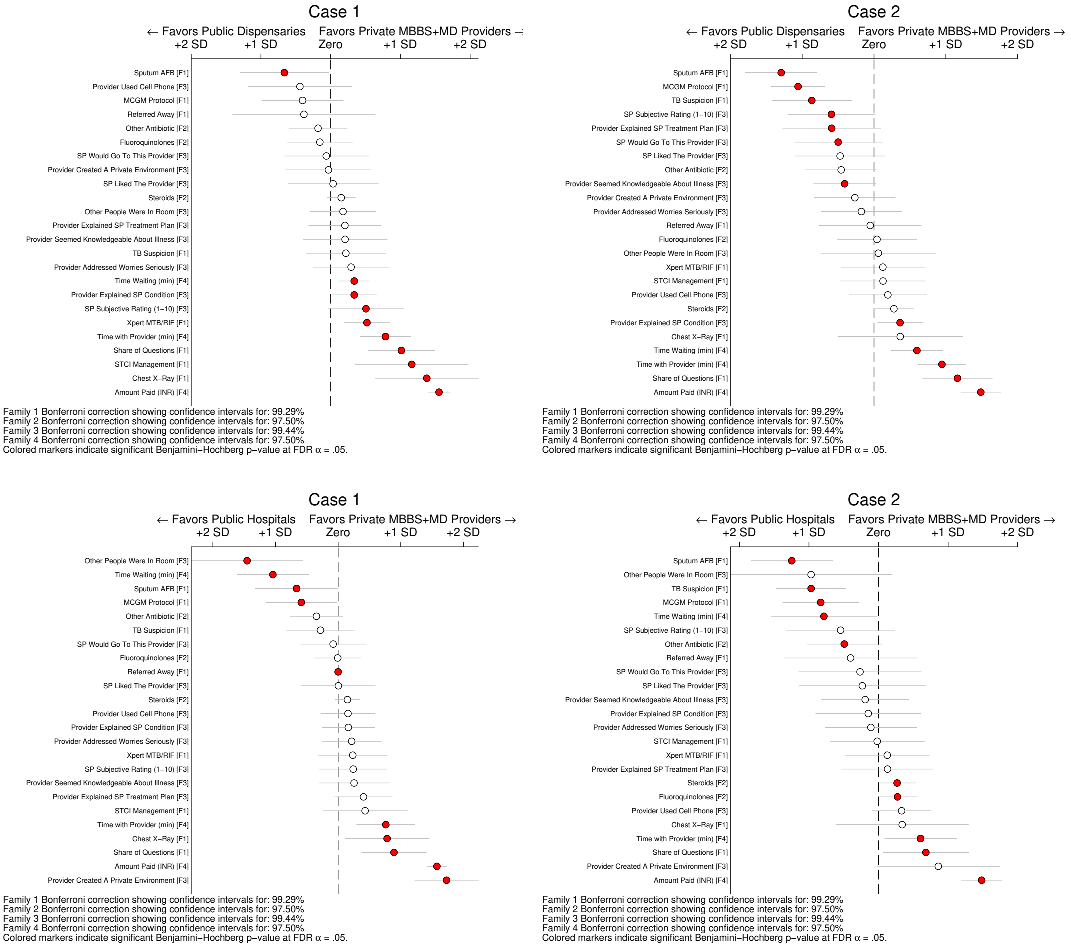

Notes: This figure reports the results of a series of regressions using both separately comparing the different types of public sector facilities with private sector MD specialist providers. Each point reports the coefficient for the indicator variable of the private sector and the corresponding confidence interval. All measures are standardized to mean 0 and standard deviation 1 for illustration purposes. Estimates are controlled for individual SP ID and the case scenario; standard errors are clustered by facility. The measures are grouped into four families: Family 1 are the appropriate clinical management outcomes; Family 2 are the medication use outcomes; Family 3 are the subjective experience measures; and Family 4 are the unassociated characteristics. Each family's confidence intervals are extended to the appropriate Bonferroni interval for the number of simultaneous hypothesis; and the p-values are considered significant if they satisfy the Benjamini-Hochberg step-up procedure (including values that may not be considered significant under their own Bonferroni interval). Measures are sorted by the magnitude and direction of the estimated coefficient.

Figure A4. Time dimensions of interactions

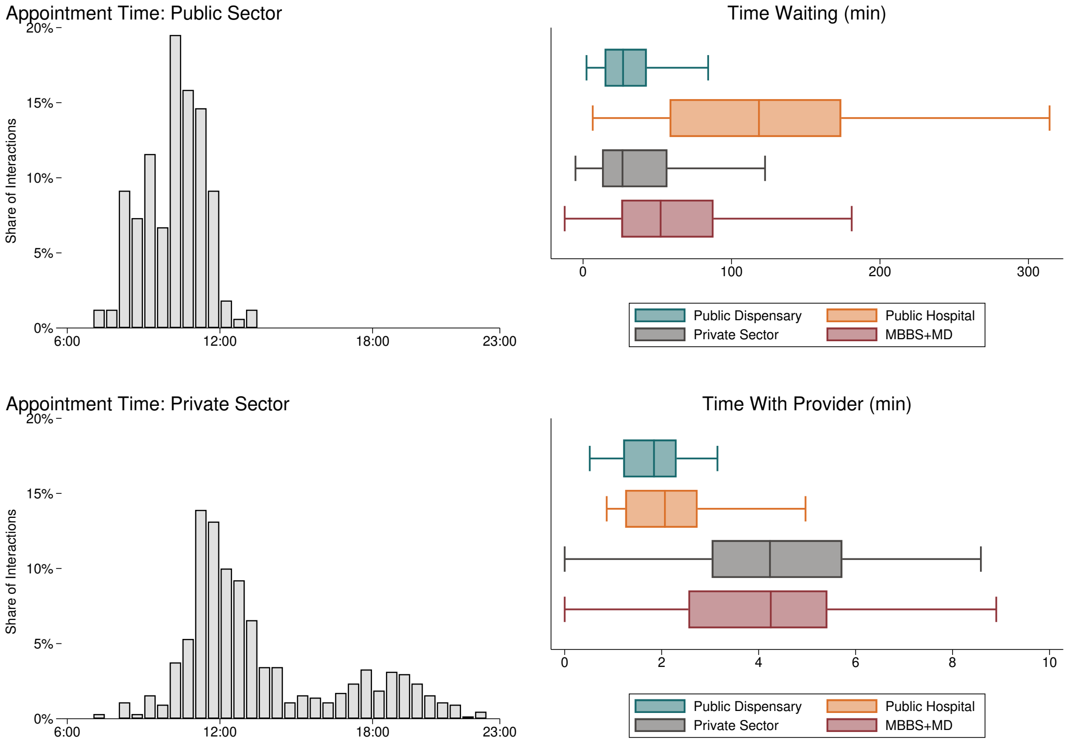

Notes: This figure summarizes temporal characteristics of interactions with various types of providers for illustration purposes. The left panels display the time of day of all interactions that SPs were able to successfully complete in each sector. Histogram bars each cover a half-hour time interval and are reported as the percentage of interactions occurring in each time slot. The right panels summarize the time waiting for each interaction and the time with each provider (in minutes), presented as a box plot with the 5th, 25th, 50th, 75th, and 95th percentiles marked.

Figure A5. Public sector sample and administrative data

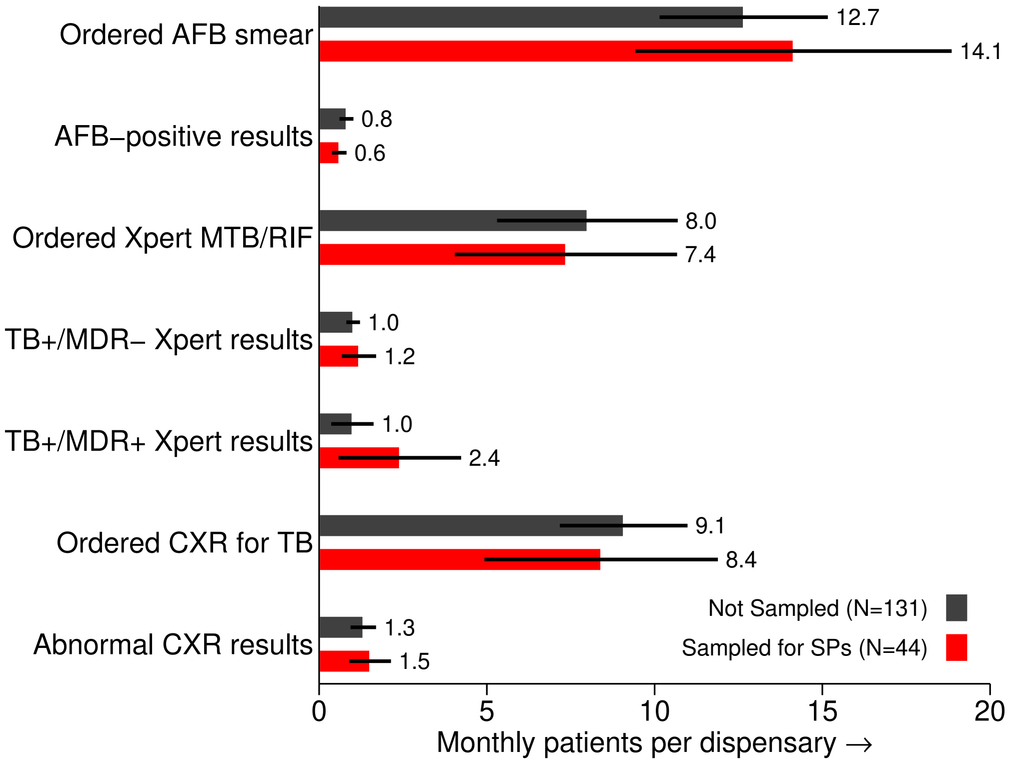

Source: Municipal Corporation of Greater Mumbai.

Daniels B, *et al. BMJ Global Health* 2022; 7:e009657. doi: 10.1136/bmjgh-2022-009657
